# Nanoarchitectonics with cetrimonium bromide on metal nanoparticles for linker-free detection of toxic metal ions and catalytic degradation of 4-nitrophenol

**DOI:** 10.3762/bjnano.15.106

**Published:** 2024-11-04

**Authors:** Akash Kumar, Raja Gopal Rayavarapu

**Affiliations:** 1 Nanomaterial Toxicology Laboratory, Drug and Chemical Toxicology Group, Food, Drug and Chemical, Environment and Systems Toxicology (FEST) Divison, CSIR-Indian Institute of Toxicology Research (CSIR-IITR), Vishvigyan Bhawan, 31 Mahatma Gandhi Marg, Lucknow 226001, India; 2 Academy of Scientific and Innovative Research (AcSIR), Ghaziabad 201002, India

**Keywords:** catalysis, CTAB, heavy metal, nanoparticles, 4-nitrophenol, sensing

## Abstract

Heavy metal ions and organic pollutants, such as 4-nitrophenol (4-NP), pose significant environmental and human health threats. Addressing these challenges necessitates using advanced nanoparticle-based systems capable of efficient detection and degradation. However, conventional approaches utilizing strong capping agents like cetrimonium bromide (CTAB) on nanoparticles lead to limitations due to the rigid nature of CTAB. This restricts its utility in heavy metal detection and 4-NP degradation, requiring additional surface modifications using linker molecules, thereby increasing process complexity and cost. To overcome these limitations, there is a critical need for the development of an easy-to-use, dual-functional, linker-free nanosystem capable of simultaneous detection of heavy metals and efficient degradation of 4-NP. For enabling linker-free/ligand-free detection of heavy metal ions and catalytic degradation of 4-NP, CTAB was engineered as a versatile capping agent on gold and silver nanoparticles. Various factors, including nanoparticle characteristics such as shape, size, metal composition, centrifugation, and NaOH amount, were investigated for their impact on the performance of CTAB-capped nanoparticles in heavy metal detection and 4-NP degradation. CTAB-Au nanospheres demonstrated limited heavy metal ion detection capability but exhibited remarkable efficiency in degrading 94.37% of 4-NP within 1 min. In contrast, silver nanospheres effectively detected Hg^2+^, Cu^2+^, and Fe^3+^ at concentrations as low as 1 ppm and degraded 90.78% of 4-NP within 30 min. Moreover, anisotropic gold nanorods (CTAB-AuNR1 and CTAB-AuNR2) showed promising sensing capabilities towards Cu^2+^, Cr^3+^, and Hg^2+^ at 0.5 OD, while efficiently degrading 4-NP within 5 min at 1 OD. This study emphasizes the importance of tailoring parameters of CTAB-capped nanoparticles for specific sensing and catalytic applications, offering potential solutions for environmental remediation and human health protection.

## Introduction

Metal nanoparticles are widely used for a great number of applications owing to their excellent optochemical properties and high surface-to-volume ratio in comparison to bulk materials. Noble metal (gold and silver) nanoprobes are emerging as versatile colorimetric and spectrophotometric nanosensors for rapid detection/degradation of heavy metal ions and toxic pollutants that pose a serious challenge to environment and human health.

Globally, acceleration of industrial growth and urbanization led to the increased release of pollutants into the environment, causing health concerns to humans. Untreated industrial effluents are released, and most heavy metal ions accumulate in water higher than the permissible limits, pollute drinking water, and are non-biodegradable. Heavy metal ions are potentially carcinogenic, and it is hard to predict their toxicity at early stages of exposure. In addition, industrial wastewater may contain toxic compounds such as the widely used 4-nitrophenol [1]. Hence, one need is to develop a dual-functional and flexible linker-free metal nanoparticle-based sensor that is rapid and affordable for the detection of heavy metal ions as well as for the degradation of organic pollutants such as 4-nitrophenol.

Developing a robust sensing platform based on metal nanoparticles requires a modulation of the surface chemistry, which is governed by the choice of capping agents on the metal surface and further dominates functionalization. Various capping agents such as citrate, PVP, and surfactants have been the choice for metal nanoparticles. Controlled size, shape, and surface properties have been achieved using strong capping and reducing agents. Capping agents maintain size, shape, and stability of the nanoparticles, and suitable capping agents can modulate the nanoarchitectonics of the nanoparticles from atomic to molecular levels [2]. The surface capping can also influence the surface properties of the nanoparticles, making them compatible with specific environments or functional groups [3].

Surfactants bind to metal surfaces and create a stable colloidal solution by preventing the nanoparticles from aggregation or clustering [4]. CTAB is a widely used cationic surfactant that provides nanoparticle ionic stability and anisotropy [5]. Although CTAB delivers high strength to nanoparticles, the use of such nanoparticles is limited in sensing, catalysis, and biomedical applications because of post-synthesis functionalization, morphology, and toxicity [6–8]. CTAB is a resilient molecule on the nanoparticle surface because of its micellar structure and does not allow for interactions with ligands via ion–ion interactions. Therefore, multiple surface modifications or linkers must be used for selective interaction between ligand and CTAB-capped gold and silver nanoparticles [7,9].

Contaminants in form of heavy metals and pollutant such as 4-nitrophenol are both dangerous to humans [10–11]. Contamination with toxic heavy metal ions, including zinc, arsenic, aluminium, chromium, iron, cobalt, copper, nickel, mercury, cadmium, and lead, causes significant chronic damage to organ systems, beginning at the cellular level [12]. Toxic heavy metal ions such as Hg^2+^ are poisonous environmental pollutants that cause damage at the DNA level by inhibiting DNA replication and DNA polymerase activity, ultimately affecting normal cell synthesis [13]. The less toxic Fe^3+^ is an essential nutrient for human health in a lower dose, while increasing the dose also leads to undesirable side effects such as iron overload (hemochromatosis) and acute iron poisoning [14]. Similarly, other metals, including copper and cobalt, significantly damage different body parts above a threshold limit. Heavy metal ions pose a severe risk to human and environmental health [15]. Besides heavy metals, 4-nitrophenol is widely used for dye synthesis, insecticides and pesticides, indicators, and photographic chemicals [16]. Regarding the use of 4-nitrophenol, there are several toxicity concerns via different exposure routes, including dermal, oral, and inhalation [17]. Therefore, developing methods to detect heavy metals at the low concentrations usually found in environmental samples is crucial. Similarly, the removal of 4-nitrophenol from exposed site requires necessary steps.

Heavy metals are detected using high-throughput techniques, such as atomic absorption spectroscopy, atomic emission spectroscopy, surface-enhanced Raman scattering, electrochemical, fluorescence, and colorimetric methods [18–19]. Catalytic hydrogenation is the preferred method for the conversion of 4-nitrophenol to 4-aminophenol, which is less toxic [20]. However, the conversion process is laborious and requires precious metals such as palladium and platinum as catalysts [20]. Colorimetric detection methods have attracted attention because of their advantages, including simplicity, sensitivity, visualization, and real-time detection, while other methods often require expensive instrumentation and complex operational procedures [21]. Colorimetric detection of heavy metals and catalytic conversion of 4-nitrophenol can be achieved using CTAB-capped gold or silver nanoparticles because of their unique surface plasmon resonance (SPR) properties, allowing for a colorimetric analysis through a change in absorption wavelength. The color of gold and silver nanoparticles highly depends on shape, size, and pH value, which are directly influenced by the ligand–metal interaction [22]. Another essential factor is surface capping, which provides colloidal stability and the surface for ionic interaction with ligands [23]. Previously, post-synthesis surface-modified CTAB-capped gold and silver nanoparticles were used to detect various compounds, including heavy metals [9,24]. Moudgil et al. showed that that poly-ʟ-lysine-coated CTAB-AgNPs are selective and sensitive for detecting Hg^2+^ [9]. GSH-modified isotropic and anisotropic CTAB-AgNPs were used to detect cobalt ions. The author found that only rod-shaped particles enabled visual detection of cobalt [25]. Yagyu et al. showed the efficient removal of radioactive cesium using Prussian blue [26]. Also, 4-nitrophenol was degraded using CTAB-capped gold nanoparticles within 60 min of the reaction in the presence of thiosulfate [27]. The study concluded that nanoparticles of 13 nm can efficiently catalyze the reaction from 4-nitrophenol to 4-aminophenol [27].

A number of studies confirmed that linker-dependent detection and catalytic performance were available using CTAB-capped gold or silver nanoparticles. Unfortunately, only very few studies described CTAB-capped nanoparticles as detection and catalytic systems without surface modifications, linkers, or buffers because of the strong binding of CTAB on the nanoparticle surface. Designing CTAB-capped gold and silver nanoparticles without linker/buffer for catalysis and detection heavy metals is a current need. In this work, we have developed a linker-free nanosensor with CTAB as capping agent on both isotropic and anisotropic gold and silver nanoparticles. The CTAB-capped metal nanoparticles could sense heavy metal ions and also helped in the rapid catalytic degradation of 4-nitrophenol.

## Materials and Methods

### Materials

Cetrimonium bromide (CTAB) (Cat No. 52370), sodium borohydride (NaBH_4_) (Cat No. 480886), and ascorbic acid (AA) (Cat No. A7506) were purchased from Sigma-Aldrich. Silver nitrate (AgNO_3_) (Cat No. 1.93200.0027) and hydrochloric acid (HCl) (Cat No. 1.93001.2521) were obtained from Merck. Sodium hydroxide (NaOH) (Cat No. TC1460), chloroauric acid trihydrate (HAuCl_4_·3H_2_O) (Cat No.10724SG001) and 4-nitrophenol (4-NP) (Cat No. 144956) were purchased from CDH fine chemicals, Finar and SRL chemicals, respectively. National Institute of Standard and Technology grade metal standards (1000 ppm) of arsenic (As^3+^), aluminum (Al^3+^), cadmium (Cd^2+^), zinc (Zn^2+^), mercury (Hg^2+^), nickel (Ni^2+^), copper (Cu^2+^), chromium (Cr^3+^), lead (Pb^2+^), iron (Fe^3+^), and cobalt (Co^2+^) were purchased from CDH Fine Chemicals, India. The chemicals obtained were used without further purification. All glassware was cleaned with aqua regia and rinsed with double distilled (DD) water before use.

### Cetrimonium bromide as capping agent

CTAB-AgNS (silver nanospheres) and CTAB-AuNS (gold nanospheres) were synthesized via wet chemical synthesis using slightly modified protocols [28–29]. In a typical synthesis carried out at 80 °C, 0.5 mL of AgNO_3_ (100 mM) was reduced using 1 mL of 100 mM NaBH_4_ in the presence of 5 mL DD water premixed with 1 mL of 100 mM cetrimonium bromide (CTAB) under stirring at 800 rpm. Similarly, gold nanospheres were synthesized by adding 0.5 mL of 25 mM gold chloride solution to 5 mL of cetrimonium bromide (100 mM) followed with the addition of 0.5 mL NaBH_4_ (100 mM) under stirring at 800 rpm. The silver and gold nanospheres were centrifuged at 18000*g* and 20000*g* for 20 min and 60 min, respectively, to remove impurities. The supernatants were discarded and the pellets were dispersed in DD water and stored at room temperature.

### Tunable aspect ratio of gold nanorods capped with CTAB

Gold nanorods of different lengths were synthesized using a two-step protocol as described in previous reports with slight modifications [30]. Briefly, in the first step, gold seeds were prepared by mixing CTAB, HAuCl_4_·3H_2_O and NaBH_4_. The seed solution acts as an initiator for synthesizing gold nanorods. In the second step, the growth solution was prepared, consisting of CTAB, AgNO_3_, HAuCl_4_·3H_2_O, HCl, and ascorbic acid. A typical synthesis involved the synthesis of CTAB-capped Au seeds of less than 4 nm. Addition to the growth solution in step 2 resulted in the formation of gold nanorods. The seeds were prepared using 200 µL of HAuCl_4_·3H_2_O (25 mM) with 2 mL of 0.1 M CTAB at 80 °C, followed by 800 µL freshly prepared ice-cold NaBH_4_ (10 mM) added under continuous stirring (800 rpm). The Growth solution of 5 mL of CTAB (100 mM) was placed on a hot plate (80 °C), 500 μL of HAuCl_4_·3H_2_O (10 mM), 50 μL/120 μL (for shorter and longer nanorods, respectively) AgNO_3_ (10 mM), 200 μL of HCl (1 M), and 80 μL of AA (100 mM) were sequentially added to the reaction mixture. Finally, 50 μL of freshly prepared seed solution was added to the growth solution to initiate the synthesis of gold nanorods. The different sizes of gold nanorods were obtained by varying the volume of AgNO_3_ solution. Finally, anisotropic AuNR1 (shorter length) and AuNR2 (longer length) were centrifuged at 16,000*g* and 12,000*g* for 20 min, respectively, to remove excess of surfactant and any impurities.

### Characterization of CTAB-capped isotropic and anisotropic metal nanoparticles

The plasmonic properties of the synthesized isotropic and anisotropic metal nanostructures were measured using an Epoch2 spectrophotometer (BioTek, USA). Hydrodynamic radius and polydispersity index (PDI) were measured using dynamic light scattering (Zetasizer Nano ZS, Malvern, UK). The zeta potential measurements were conducted to determine the surface charge for both isotropic and anisotropic metal nanoparticles using zeta cuvettes (DTS0012). The precise size and morphology of CTAB-AgNS, CTAB-AuNS, CTAB-AuNR1, and CTAB-AuNR2 were observed under a transmission electron microscope (TEM, 120 kV; FEI Tecnai). The nanoparticles were placed onto 200-mesh carbon-coated copper grids. The average size of the nanoparticles was calculated using ImageJ software (USA). The crystal structure of CTAB-capped gold and silver nanoparticles was determined using XRD (Rigaku Smartlab, Japan) in a 2θ range of 35° to 80°. The nanoparticle solutions were air-dried, and the obtained powders (20 mg) were used for measurement. The functionalization of AgNS, AuNS, AuNR1, and AuNR2 with CTAB was validated through Fourier-transform infrared spectroscopy (Thermo Scientific, Nicolet iS5, USA). 10 mg of the air-dried nanoparticles were placed over a diamond and measured in the range of 400–4000 cm^−1^.

### Quantification of CTAB through UHPLC

The quantification of bound CTAB on the nanoparticles’ surface was analyzed using ultrahigh-performance liquid chromatography (UHPLC; Shimadzu, Nexera, Tokyo, Japan). The chromatography system equipped with an SPD-M20A photodiode array (PDA) detector with a wavelength range of 190–800 nm, a degreaser (DGU-20A_5R_), autosampler (SIL-30AD), and quaternary pump (LC-30AD). The separation peak for CTAB was analyzed using HpersilGold^TM^ C18 (100 × 2.1 mm, 10 μm) with a pore diameter of 1.9 μm. The injection volume was 5 µL, and the wavelength was 208 nm for CTAB quantification. The mobile phase included a mixture of acetonitrile and water (40:60 v/v) at a 0.2 mL/min flow rate. The method was developed using CTAB powder as standard at different concentrations of 0.625, 1.2, 2.5, 5, 10, and 20 µg/mL in water. The blank sample (which consisted only of solvent) was also analyzed to confirm the peak value of the compound. The supernatants of centrifuged CTAB-capped nanoparticles were used for CTAB quantification.

### Stability of CTAB-capped metal nanoparticles in NaCl and NaOH solutions

In order to determine the ability and functionality of CTAB on metal nanoparticle surfaces, we have studied their stability in commonly used 1 M solutions of sodium chloride and sodium hydroxide. The stability of as-prepared and centrifuged nanoparticles of CTAB-AgNS, CTAB-AuNS, CTAB-AuNR1, and CTAB-AuNR2 were evaluated. The optical density (OD) of all CTAB-capped nanoparticles was kept in the vicinity of 0.5 ± 0.1 and 1.0 ± 0.1. Aliquots of 1 M NaOH (volumes ranging between 5–50 μL) was added to the nanoparticle solutions. Also, CTAB-AgNS, CTAB-AuNS, CTAB-AuNR1, and CTAB-AuNR2 were added to 1 M NaCl solution while keeping the optical density of the nanoparticles constant at 0.5 and 1.

### Linker-free sensing of heavy metal ions

National Institute of Standard and Technology grade metal standards (1000 ppm) of As^3+^, Al^3+^, Cd^2+^, Zn^2+^, Hg^2+^, Ni^2+^, Cu^2+^, Cr^3+^, Pb^2+^, Fe^3+^, and Co^2+^ were diluted to a concentration of 10 ppm. A uniform concentration of nanoparticles in terms of optical density (OD) was maintained throughout the experiment. The concentration of CTAB-AgNS, CTAB-AuNS, CTAB-AuNR1, and CTAB-AuNR2 was kept at constant values of 0.5 ± 0.1 OD and 1.0 ± 0.1 OD per milliliter of water during the test. In a typical sensing experiment, the corresponding metal ions were spiked in water. Then, CTAB-AgNS, CTAB-AuNS, CTAB-AuNR1, and CTAB-AuNR2 were dispersed and examined regarding colorimetric/spectrophotometric changes. CTAB-AgNS, CTAB-AuNS, CTAB-AuNR1, and CTAB-AuNR2 were used with aliquots of NaOH (1 M) in a volume range of 5–50 μL. The pH of a sample was not measured because a volume of 5 μL NaOH already yielded pH 12. Consequently, we chose the volume of NaOH as a parameter instead of the pH value.

### Catalytic degradation of 4-nitrophenol

The catalytic activity of CTAB-AgNS, CTAB-AuNS, CTAB-AuNR1, and CTAB-AuNR2 was determined by reducing 4-nitrophenol (4-NP) to 4-aminophenol (4-AP) in the presence of NaBH_4_. All reactions were carried out at room temperature in a 3.5 mL quartz cuvette. The reagent for catalysis was added in a sequence of 2 mL of 4-nitrophenol (0.1 mM), 1 mL of NaBH_4_ (100 mM), and 50 µL (0.5 ± 0.1 OD and 1.0 ± 0.1 OD) of CTAB-AgNS, CTAB-AuNS, CTAB-AuNR1, and CTAB-AuNR2, centrifuged and as-prepared, respectively. The catalytic conversion of 4-NP to 4-AP was measured using a UV–vis spectrophotometer at 300–900 nm. The disappearance of the color upon adding nanoparticles was measured and further, the change in the plasmon band was recorded. As controls, we chose bare nanospheres of gold/silver and compared them to the CTAB-capped metal nanoparticles [31].

## Results and Discussion

**Figure 1:**
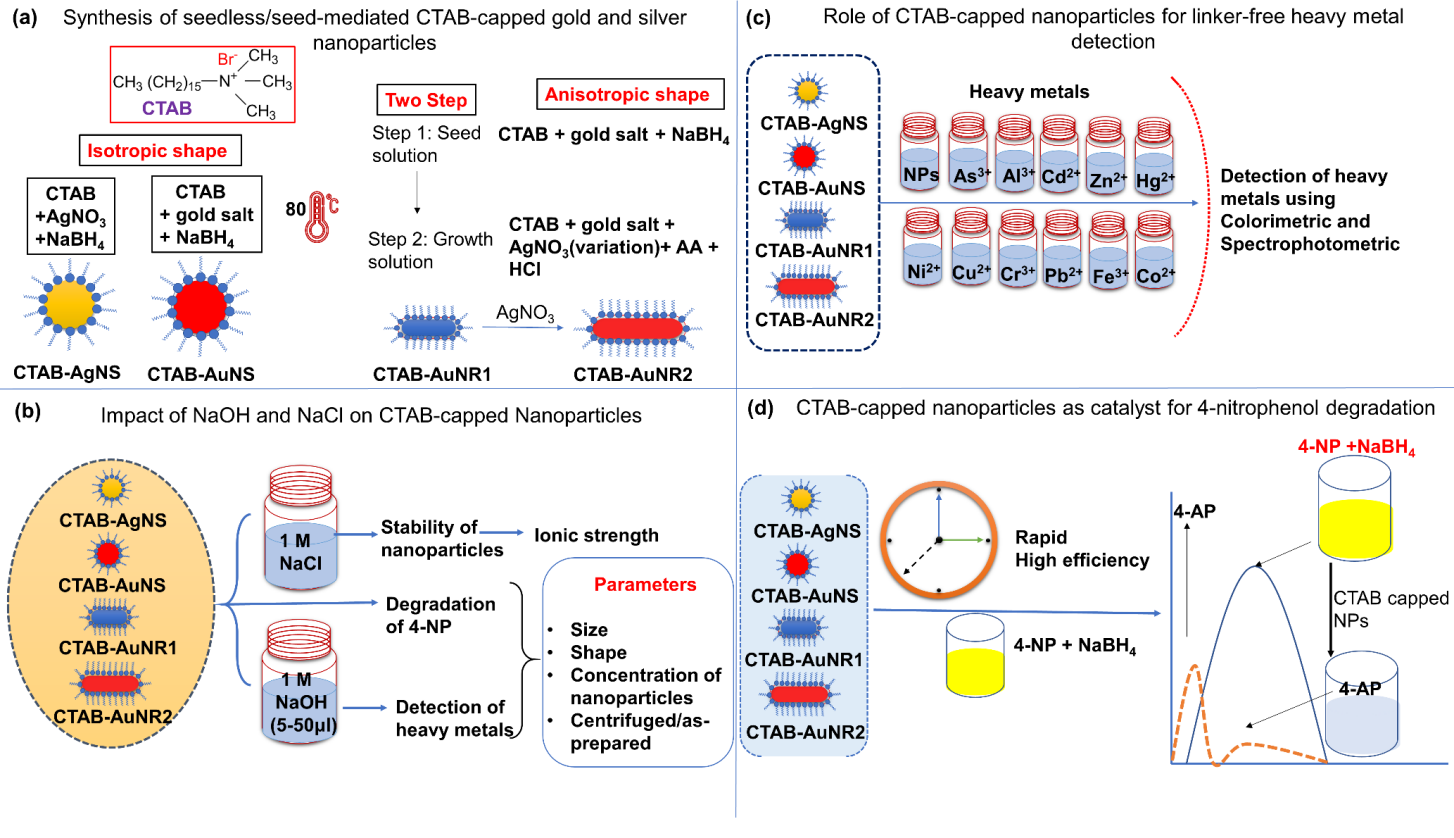
(a) Wet chemical synthesis of CTAB-capped isotropic gold and silver as well as anisotropic gold nanoparticles through seedless and seed-mediated approaches. (b) Assessment of nanoparticle stability in 1 M NaCl and NaOH for sensing applications. Evaluation of linker-free CTAB-capped metal nanoparticles for (c) sensing heavy metal ions and (d) the catalytic degradation of 4-nitrophenol.

Figure 1a shows the synthesis of isotropic and anisotropic metal nanoparticles using CTAB as surfactant. Silver and gold nanospheres, along with nanorods of different lengths were synthesized using wet chemical reduction, as shown in Figure 1a. A unique feature of CTAB is its robust and selective binding to certain crystal facets of metal surfaces that define the growth and nucleation of nanoparticles. CTAB on metal surfaces plays a key role in nanoparticle stabilization but hinders many applications, which in some instances require an external linker or ligand for usable applications. It was reported that for CTAB-capped nanoparticles to enable sensing, the use of a linker on CTAB was mandatory for heavy metal ion detection. Linker-free detection of heavy metals using CTAB is a challenge and pre-requisite for enabling faster and facile approaches. In this work, we designed several strategies involving the desorption of CTAB from the metal surface in the presence of solvents such as NaOH, which can tune the amount of CTAB on the surface. A recent study showed that sodium borohydride removed CTAB from metal surfaces for ligand exchange, which helped in the detection of heavy metal ions [32]. The impact of NaOH and NaCl was assessed regarding the stability of the synthesized metal nanoparticles at extreme concentrations (Figure 1b). The addition of 1 M NaOH at various volumes (5–50 µL) results in the controlled desorption of CTAB from the metal surface. A recent study demonstrated the ability of NaOH to interfere with CTAB micelles, where the pH value played a key role [33]. Optimized conditions enabled the linker-free sensing of heavy metal ions (Figure 1c). Nanoparticles dispersed in DD water were chosen as control (represented as NPs in Figure 1c). Additionally, CTAB-capped gold and silver nanoparticles have been reported for their enhanced catalytic activity. The simplest and fastest method for degradation or catalytic conversion of 4-nitrophenol is using a strong reducing agent (such as NaBH_4_) in the presence of a catalytic agent (i.e., the nanoparticles) [27]. In the current study, we also examined the catalytic properties of CTAB-capped nanoparticles in the degradation of 4-nitrophenol (Figure 1d). The catalytic properties of CTAB-capped nanoparticles highly depend on various factors, including size, shape, and reaction parameters [34]. However, there was a gap in the literature regarding which factor is most crucial to the efficient degradation of 4-nitrophenol using nanoparticles. Some studies suggest size is essential, while others suggest pH, surface capping, and linker molecules are required to degrade 4-nitrophenol. Therefore, this study analyzed the impact of size, shape, metal type, and nanoparticle concentration on converting 4-nitrophenol to 4-aminophenol.

### Characterization of CTAB-capped nanoparticles

#### UV–vis, DLS, Zeta, FTIR, XRD, and TEM analyses

**Figure 2:**
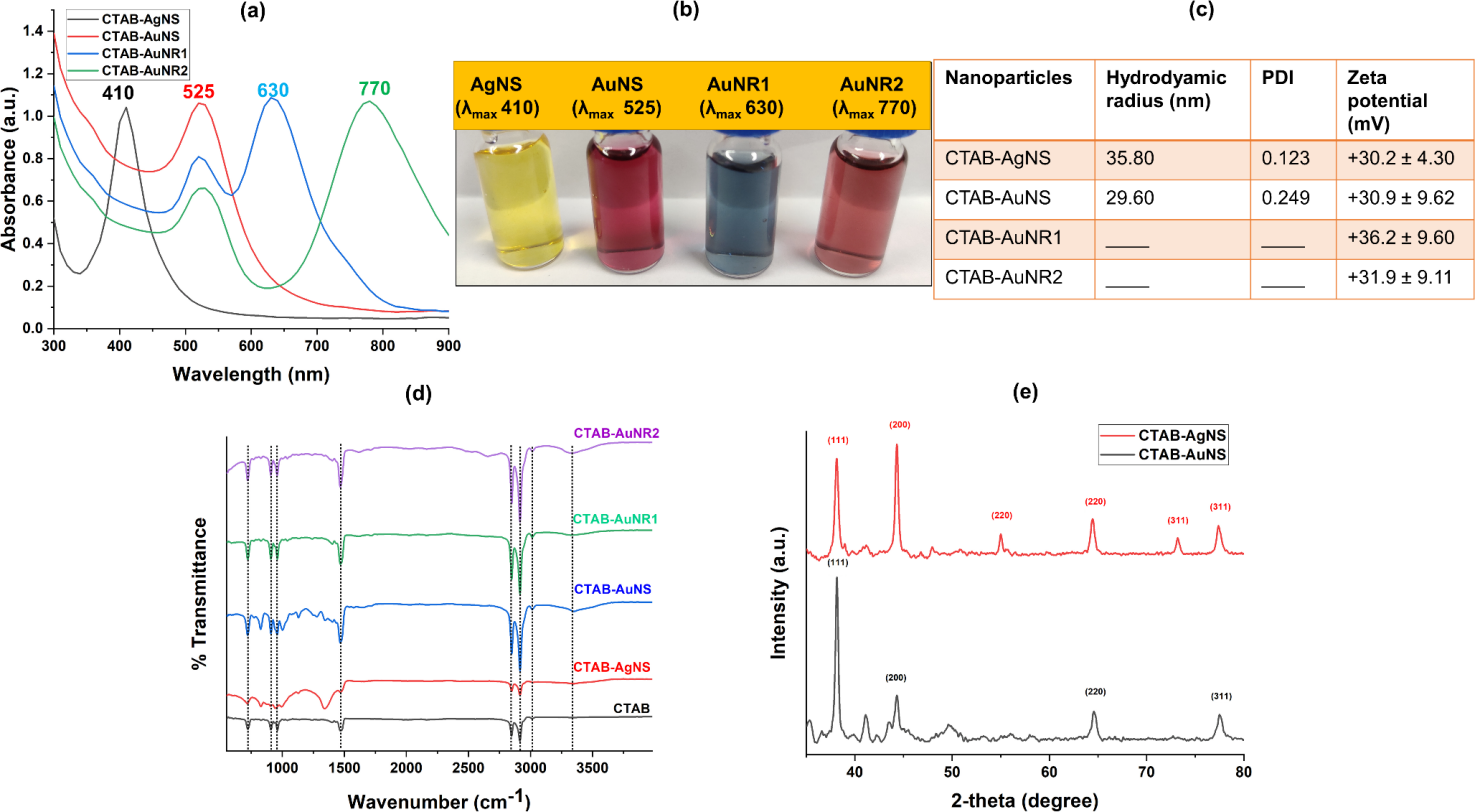
(a) Optical absorbance of CTAB-AgNS, CTAB-AuNS, CTAB-AuNR1, and CTAB-AuNR2 with maximum absorption wavelengths (λ_max_) of 410, 525, 630, and 770 nm, respectively. (b) Colloidal solution of CTAB-capped nanoparticles. (c) Hydrodynamic size and zeta potential. (d) FTIR spectra and (e) XRD patterns of CTAB-capped silver and gold nanoparticles.

Physicochemical characterization was performed using optical spectroscopy, DLS, FTIR, XRD, and TEM analyses. Figure 2a shows the synthesized isotropic silver and gold nanospheres with plasmon bands at 410 nm (AgNS) and 525 nm (AuNS). The anisotropic tunable gold nanorods with longitudinal plasmon bands at 630 nm (AuNR1) and 770 nm (AuNR2) indicate the formation of different lengths of nanorods.

The colloidal solutions of the metal nanoparticles having different sizes and shapes resulted in different colors (Figure 2b), a characteristic feature of gold and silver nanoparticles involving the change in surface plasmon resonance of the metal nanostructures. A single absorbance peak correlates to the symmetrical shape and collective oscillation of free electrons on the nanoparticle surface. This phenomenon is known as localized surface plasmon resonance (LSPR), a characteristic feature of metallic nanoparticles such as gold and silver [35]. The length of the gold nanorods influences the LSPR [36]. The CTAB molecules on the shorter gold nanorods (20.8 ± 9.5 nm) are densely packed compared to longer nanorods (36.5 ± 7.3 nm). The high curvature of the nanorods leads to enhanced stabilization due to the larger inter-micellar channels generated on the flat side facets of the gold nanorods [37]. In comparison, the CTAB molecules on the longer nanorods are scattered over the side faces of the nanorods, which may lead to aggregation. Changes in pH or ionic strength are also capable of decreasing the CTAB binding to surfaces of longer gold nanorods. Therefore, longer nanorods are more prone to aggregation than shorter rods. The packing density of CTAB is also reported to be a factor that controls the size of metal nanoparticles [38]. The isotropic gold nanospheres exhibit an equal distribution of CTAB on the surfaces due to the isotropic geometry. The amount of CTAB is critical in determining the functional aspects of the metal nanospheres or nanorods.

Hydrodynamic radius, polydispersity, and surface charge of the synthesized CTAB-capped nanoparticles were measured as shown in Figure 2c. The DLS measurement indicated average hydrodynamic radii of 35.80 and 29.60 nm for isotropic silver and gold nanospheres, respectively. DLS measurements of anisotropic nanorods (AuNR1 and AuNR2) could not be carried out because of limitations of the instrument [39]. The nanospheres of silver and gold showed good PDI values of 0.123 and 0.249 for silver and gold nanospheres, respectively (Figure 2c). The very low PDIs prove that the nanospheres are monodisperse and non-aggregated. A PDI of less than 0.3 typically indicates a relatively narrow and well-controlled size distribution [40]. CTAB forms a monolayer around the nanoparticles, providing a consistent and uniform surface coverage. This uniformity in surface passivation contributes to the narrow size distribution of the particles. The long hydrocarbon tails of the CTAB molecules extending from the nanoparticle surface create steric repulsive forces between the particles. This repulsion prevents particles from getting too close to each other, thus minimizing aggregation.

The CTAB-capped AgNS, AuNS, AuNR1, and AuNR2 showed positive zeta potential values of 30.2 ± 4.3, 30.9 ± 9.6, 36.2 ± 9.6, and 31.9 ± 9.1 mV, respectively (Figure 2c). Zeta potential values beyond −30 mV and +30 mV indicate excellent colloidal stability due to strong repulsive forces among the nanoparticles [41]. The positive zeta potential values are caused by the having positively charged head groups of CTAB molecules, which are attracted to the negatively charged surfaces of the metal nanoparticles and form a cationic layer around them [28].

CTAB-AgNS, CTAB-AuNS, CTAB-AuNR1, and CTAB-AuNR2 were analyzed for functional group identification using FTIR (Figure 2d). CTAB contains a long alkyl chain that typically results in peaks at 2920 and 2850 cm^−1^ in the FTIR spectrum. These peaks correspond to the symmetric and antisymmetric stretching vibrations of alkyl C–H bonds [28]. CTAB-capped gold and silver nanoparticles exhibit peaks at the same wavenumber as the parent compound, but with different intensities (Figure 2d). The doublet at 1462 and 1472 cm^−1^ obtained from CTAB is attributed to the CH_2_ scissoring mode and indicates close-packing of the methylene chains [42]. However, for CTAB-AgNS, CTAB-AuNS, CTAB-AuNR1, and CTAB-AuNR2, the peak splits into two parts with different intensities because of crystallinity loss in the hydrocarbon regions or the morphology of the nanoparticles. The singlets at 962 cm^−1^ for parent CTAB and 946 cm^−1^ for CTAB-AgNS correspond to C–N^+^ stretching bands, and the shift in wavenumber might be due to the interaction between the N-containing amino group and the silver metal surface [28]. However, the peak did not shift in the case of gold nanoparticles (AuNS, AuNR1, and AuNR2). The characteristic [C–H–Ag] vibration was missing, indicating the absence of C–H binding to the metal surface. The new minor peaks of CTAB-capped AgNS and AuNS at 828, 1004, and 1130 cm^−1^, which are not present in parent CTAB molecules, are also considered as stretching of C–N^+^ affected by the metal surface, as described by Nikoobakht and El-Sayed for CTAB-capped gold nanoparticles [43]. The singlet at 730 cm^−1^ for parent (powder) CTAB and CTAB-capped nanoparticles is due to CH_2_ rocking. The broad band at 3390 cm^−1^ in parent CTAB corresponds to N–H stretching, which is shifted to 3315 cm^−1^ after binding to the gold and silver nanoparticles. The overlap between peaks of parent CTAB with those of CTAB-capped metal nanoparticles indicates the successful capping of the nanoparticle surface without compromising their structural property.

To determine the crystalline structure of the synthesized silver and gold nanoparticles, silver and gold nanospheres capped with CTAB were measured with XRD (Figure 2e). The most common crystal structure of gold and silver in nanoparticles is face-centered cubic (FCC). In the XRD pattern of FCC CTAB-AuNS, we observe major diffraction peaks at around 38.2°, 44.1°, 64.5°, and 77.4°, corresponding to the (111), (200), (220), and (311) planes, respectively [42]. In the case of CTAB-AgNS, we observe major peaks at 38.1°, 44.3°, 64.4°, and 77.5°, corresponding to the (111), (200), (220), and (311) planes [44]. However, we also observed two minor peaks at 55.04° and 73.14°, which correspond to the (220) and (311) planes; these were also observed in other studies with CTAB-capped silver nanoparticles [44]. These peaks might be related to the formation of AgO in the nanoparticles, which is reported elsewhere [44].

**Figure 3:**
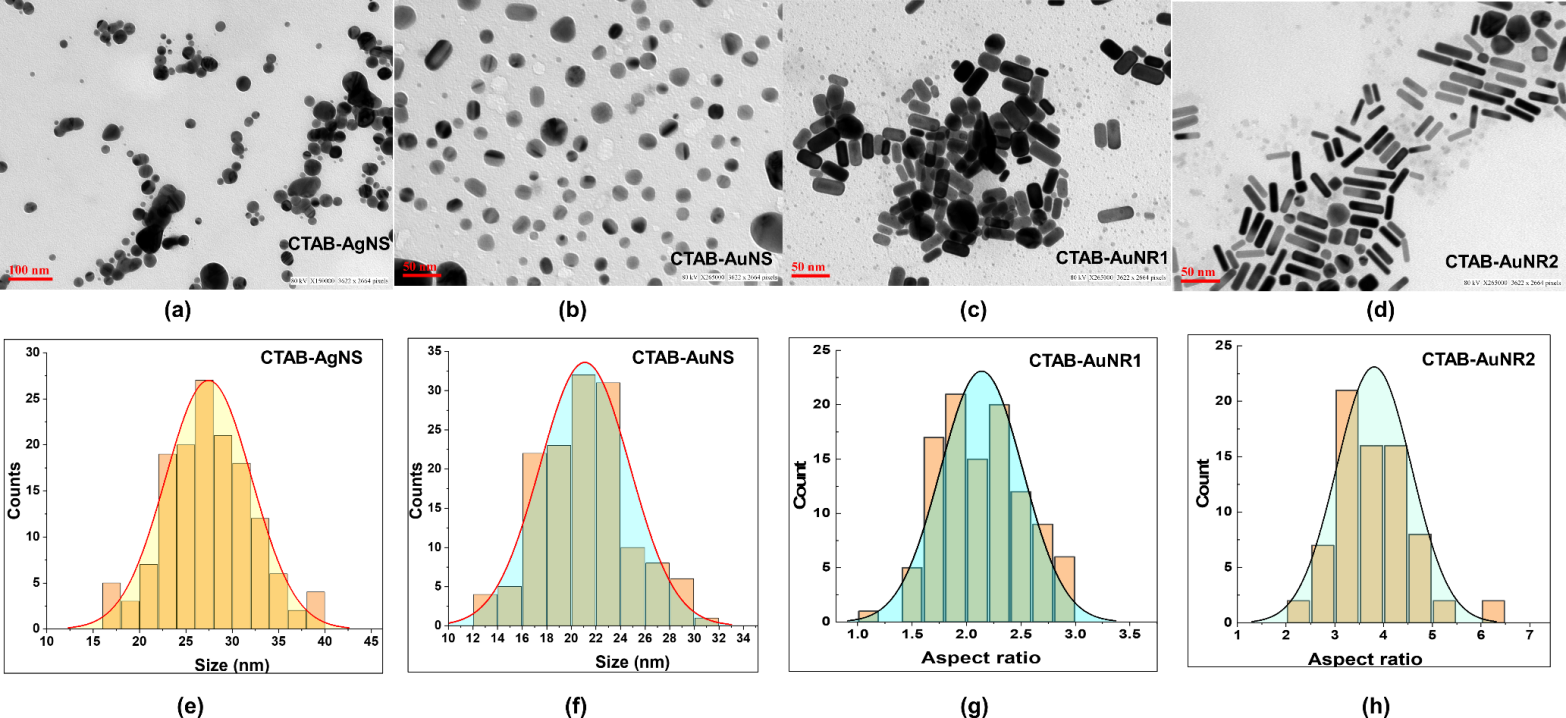
TEM images and mean size distribution of (a, e) CTAB-AgNS, (b, f) CTAB-AuNS. TEM images and aspect ratio histograms of (c, g) CTAB-AuNR1 and (d, h) CTAB-AuNR2. The average size for each sample was calculated from 100 individual nanoparticles.

Transmission electron microscopy (TEM) enables precise nanoparticle size and shape measurements. TEM images show that AuNR1 and AuNR2 have a rod-like shape with different sizes, whereas AuNS and AgNS are spherical (Figure 3a–d). The mean sizes of CTAB-AgNS and CTAB-AuNS are 27.4 ± 4.6 and 21.1 ± 3.6 nm, respectively (Figure 3e and 3f). The mean average lengths of CTAB-AuNR1 and CTAB-AuNR2 were 20.76 ± 9.5 and 36.54 ± 7.3 nm, respectively. Also, there was a change in aspect ratio from 2.1 ± 0.3 to 3.8 ± 0.7 for AuNR1 and AuNR2, respectively (Figure 3g,h). Figure 3c and 3d show different sizes of nanorods for both AuNR1 and AuNR2 with 81.4% and 82.0% population, respectively. A total of 100 nanoparticles from each sample was considered for the measurements of average size.

### Quantification of CTAB using UHPLC on nanoparticle surface

The quantity of CTAB on the nanoparticle surface was determined through an indirect approach wherein CTAB-capped metal nanoparticles underwent centrifugation, and the resulting supernatant was collected. The CTAB content in the supernatant was quantified using the developed UHPLC method. Subsequently, the remaining CTAB on the nanoparticles’ surface was calculated by subtracting the collected CTAB amount from the initial quantity provided to the reaction mixture. In UHPLC, a peak at 208 nm, with a retention time of 0.8–0.9 min, was observed in the CTAB standard, utilizing a PDA detector within a linear range of 0.625–20 µg/mL. This standard was further applied to confirm and quantify CTAB in the nanoparticle supernatant (Supporting Information File 1, Figure S1). During sample analysis, a similar peak was consistently observed in all CTAB-capped nanoparticle supernatants, appropriately diluted within the linear range. The final concentrations of CTAB in CTAB-capped AgNS, AuNS, AuNR1, and AuNR2 were found to be 5612, 30400, 31020, and 30655 µg/mL. After centrifugation, the CTAB content in the obtained supernatant was 4756, 15940, 17200, and 18340 µg/mL, as indicated in Table 1. Consequently, the CTAB remaining on CTAB-AgNS, CTAB-AuNS, CTAB-AuNR1, and CTAB-AuNR2 surfaces was determined to be 856.30, 14460, 13820, and 12315 µg/mL, respectively. Gold nanoparticles exhibit a higher amount of bound CTAB than silver nanoparticles.

**Table 1:** Quantification of CTAB using the UHPLC method.

Sample	CTAB added (µg/mL)	CTAB obtained (µg/mL)	CTAB bound on nanoparticles (µg/mL)	Binding efficiency (%)

CTAB-AgNS	5612	4756 ± 34	856.30	15.25%
CTAB-AuNS	30400	15940 ± 124	14460	47.56%
CTAB-AuNR1	31020	17200 ± 100	13820	44.55%
CTAB-AuNR2	30655	18340 ± 61	12315	40.17%

### Impact of NaCl and NaOH on CTAB-capped nanoparticles

**Figure 4:**
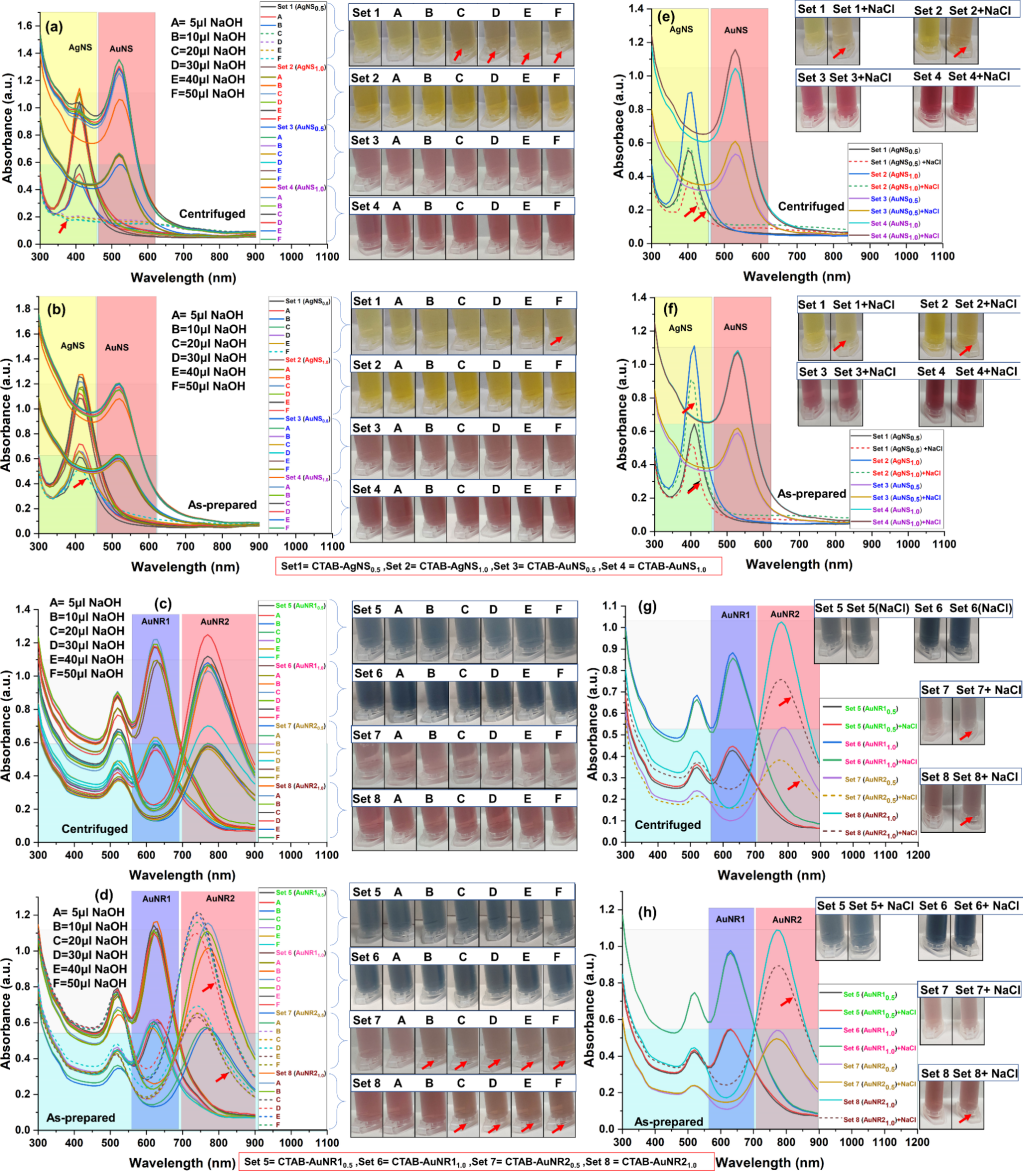
Impact of NaOH and NaCl on as-prepared/centrifuged CTAB-capped nanoparticles. (a, b) NaOH impact on centrifuged and as-prepared isotropic CTAB-capped nanoparticles (AgNS_0.5_, AgNS_1.0_, AuNS_0.5_, and AuNS_1.0_). (c, d) NaOH impact on centrifuged and as-prepared anisotropic CTAB-capped nanoparticles (AuNR1_0.5_, AuNR1_1.0_, AuNR2_0.5_ and AuNR2_1.0_). (e, f) NaCl impact on centrifuged and as-prepared isotropic CTAB-capped nanoparticles (AgNS_0.5_, AgNS_1.0_, AuNS_0.5_ and AuNS_1.0_). (g, h) NaCl impact on centrifuged and as-prepared anisotropic CTAB-capped nanoparticles (AuNR1_0.5_, AuNR1_1.0_, AuNR2_0.5_ and AuNR2_1.0_). The images attached with spectral absorbance show the color change upon adding NaOH and NaCl. The subscripts 0.5 and 1.0 represent the nanoparticle concentration in OD. Sets 1–8 correspond to nanoparticles AgNS_0.5_, AgNS_1.0_, AuNS_0.5_, AuNS_1.0_, AuNR1_0.5_, AuNR1_1.0_, AuNR2_0.5_, and AuNR2_1.0_ respectively. A, B, C, D, E, and F mean NaOH volumes of 5, 10, 20, 30, 40, and 50 μL, respectively, added to the nanoparticles. The short dashed line shows the change in the sample compared to the control (marked by an arrow). The first centrifuge tube in each colorimetric panel represented as control for that panel.

The stability and dispersibility of the CTAB-capped metal nanoparticles were assessed in 1 M NaOH and NaCl. NaOH or NaCl may lead to the desorption of CTAB from the metal surface, which plays a key role in detecting heavy metal ions. The addition of various volumes of NaOH to the metal nanoparticles of different concentrations (OD of 0.5 ± 0.1 and 1.0 ± 0.1) resulted in both colorimetric and spectrophotometric detection of heavy metal ions. The different sets 1–8 represent CTAB-AgNS, CTAB-AuNS, CTAB-AuNR1 and CTAB-AuNR2, each with 0.5 ± 0.1 and 1.0 ± 0.1 OD. As-prepared and centrifuged nanoparticles were tested to assess their stability in NaOH (5–50 μL) and NaCl. Centrifuged CTAB-AgNS_0.5_ (set 1) shows NaOH volume-dependent aggregation of the silver nanospheres (Figure 4a). The peak flattening of centrifuged AgNS_0.5_ from 20 μL NaOH (set 1, C–F) onward confirmed nanoparticle aggregation, leading to colorimetric differences compared to the control (Figure 4a).

In contrast, centrifuged AgNS_1.0_ (set 2) was highly stable in NaOH up to 50 μL (A–F) compared to AgNS_0.5_ (Figure 4a). This stability is attributed to the increased nanoparticle concentration and the amount of CTAB. A similar trend was observed in the case of as-prepared AgNS_0.5_ (set 1) and AgNS_1.0_ (set 2) (Figure 4b). However, the intensity of as-prepared AgNS_0.5_ decreases with 50 μL NaOH, evident by a color change (Figure 4b). The stability of centrifuged and as-prepared AgNS_1.0_ give a clear indication that nanoparticles with higher concentrations are stable in NaOH, which is not observed in the case of centrifuged and as-prepared AgNS_0.5_. In a study by Yadav et al., NaOH (0.1 N) induced a hyperchromic effect in silver nanoparticles, which was not observed in our case [45]. In our study, no plasmonic shift was observed, but a slight decrease in intensity at lower concentrations of silver nanoparticles might be due to the strong capping of CTAB on the nanoparticles’ surface. Apart from nanoparticle concentration, the amount of the capping agent on the surface of nanoparticles plays a significant role in providing stability, as reported elsewhere [3]. The concentration of CTAB that remained on silver nanoparticles after centrifugation was significantly reduced (Table 1). However, the role of the capping agent is not limited to stability; it also enhances the application potential of nanoparticles in the presence of NaOH. Gold nanoparticles are highly stable compared to silver nanoparticles, as silver nanoparticles are prone to oxidation [46].

Interestingly, the centrifuged/as-prepared CTAB-AuNS_0.5_ (set 3) and AuNS_1.0_ (set 4) showed no plasmonic change upon the addition of different volumes of NaOH (Figure 4a,b). Also, no significant difference in the color of AuNS was observed (Figure 4a,b). This might be because the amount of CTAB on the gold nanospheres is significantly higher than on silver nanoparticles in both centrifuged and as-prepared samples (Table 1). This suggests that the nature of the metal and the strong capping in nanoparticles are also crucial for their enhanced stability in NaOH. In addition, the shape-dependent impact of nanoparticle stability in NaOH was evaluated using gold nanorods (AuNRs) of two different lengths.

AuNR1_0.5_ (set 5), AuNR1_1.0_ (set 6), AuNR2_0.5_ (set 7), and AuNR2_1.0_ (set 8) showed no plasmonic and colorimetric changes in the presence of NaOH (Figure 4c). The as-prepared AuNR2_0.5_ and AuNR2_1.0_ showed a blueshift of plasmons along with a color change in the presence of more than 10 μL and 20 µL NaOH, respectively. In contrast, AuNR1 at both OD did not show any significant changes (Figure 4d). These results revealed that longer gold rods in the as-prepared state show a blueshift of plasmons due to the easy desorption of CTAB in the presence of NaOH, which is not observed in the case of small gold nanorods and nanospheres. The CTAB is tightly packed on short nanorods and nanospheres [43]. It was reported that NaOH significantly alters the micelles of quaternary ammonium surfactants [47]. This ultimately affects the capping ability of CTAB, thus providing a platform for interaction with the ligand. Furthermore, the nanoparticles designed for sensing applications should have higher ionic strength, which provides them the capability for heavy metal sensing in real scenarios. To determine the ionic strength of the synthesized nanoparticles, their stability in ionic salts, such as NaCl, must be evaluated. Centrifuged/as-prepared CTAB-AgNS_0.5_ and AgNS_1.0_ show a decrease in the plasmonic intensity of nanoparticles and a change in color in the presence of NaCl (Figure 4e,f). However, no flattening or aggregation of silver nanoparticles was noticed (Figure 4e,f). The decrease in the intensity of silver nanoparticles might be due to less CTAB on the nanoparticles’ surface or a decrease in CTAB CMC value. As reported elsewhere, the reduction in CTAB CMC is associated with NaCl concentration [48]. Centrifuged and as-prepared AuNS_0.5_ and AuNS_1.0_ showed no absorbance or colorimetric change in the presence of NaCl (Figure 4e,f).

Similarly, centrifuged and as-prepared AuNR1s (AuNR1_0.5_ and AuNR1_1.0_) showed no changes in plasmon frequency or color of the nanoparticles in the presence of NaCl (Figure 4g,h). Although centrifuged and as-prepared AuNR2_0.5_ and AuNR2_1.0_ showed a noticeable difference in the absorption spectrum compared to the control (Figure 4g,h). Previously, several reports confirmed the use of NaOH and NaCl in nanoparticle synthesis or surface modification for different applications. This experimental study demonstrated that CTAB-capped isotropic and anisotropic nanoparticles are stable in NaOH and NaCl and will be used for further sensing applications. In comparison with other studies, the synthesized nanoparticles are stable at higher concentrations of NaCl and NaOH. The colloidal stability of nanoparticles represents the initial step towards designing them as environmental heavy metal detection probes.

### Detection of heavy metals

#### Isotropic CTAB-capped AgNS and AuNS

**Figure 5:**
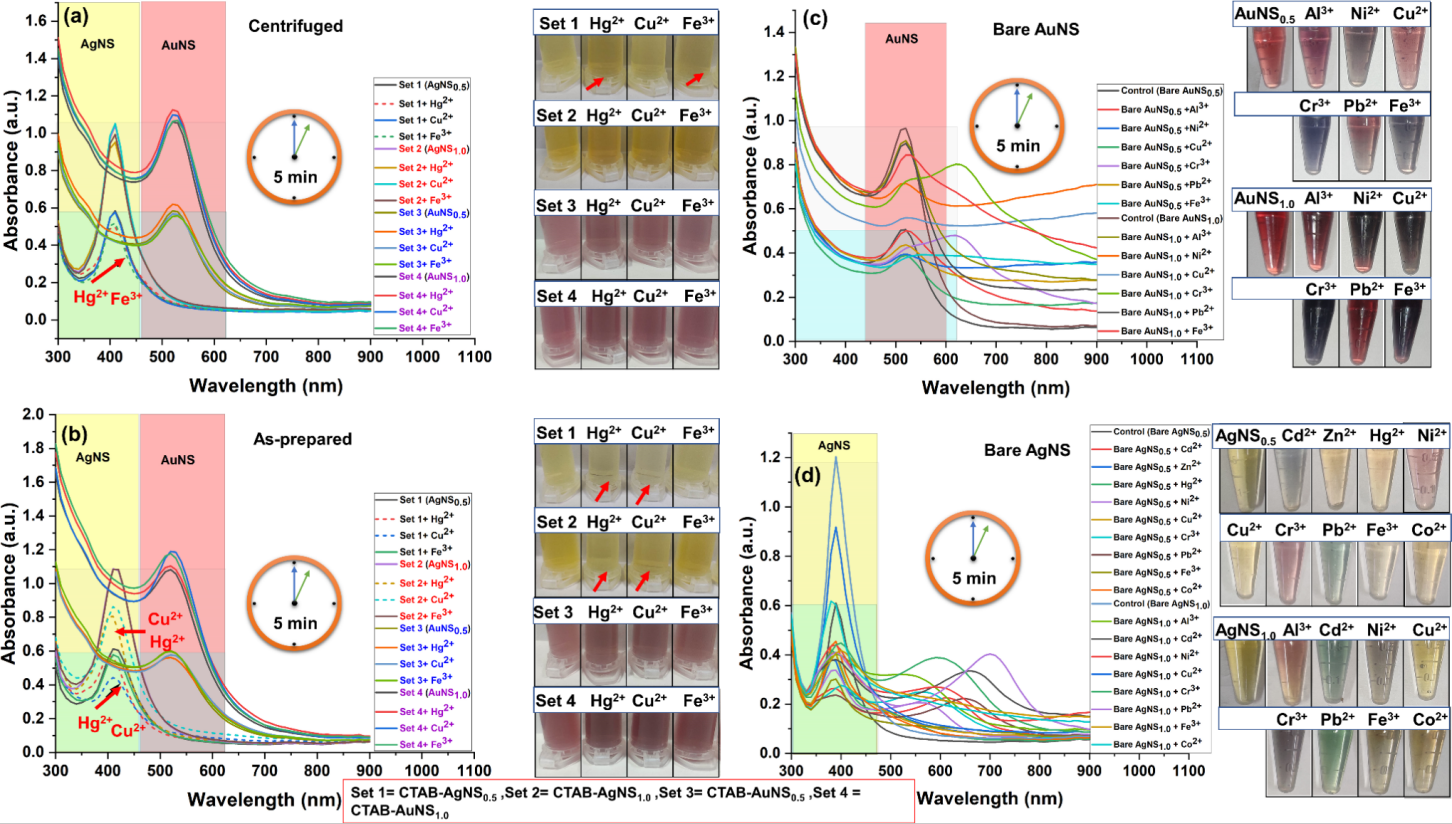
Spectrophotometric and colorimetric detection of heavy metals using centrifuged/as-prepared isotropic metal nanoparticles in the absence of NaOH. (a) Detection of Hg^2+^ and Fe^3+^ using centrifuged AgNS_0.5_, while AgNS_1.0,_ AuNS_0.5_, and AuNS_1.0_ did not detect the metals. (b) Detection of Hg^2+^ and Cu^2+^ using as-prepared AgNS_0.5_ and AgNS_1.0_, while AuNS_0.5_, and AuNS_1.0_ did not detect the metals. (c) Detection of Al^3+^, Ni^2+^, Cu^2+^, Cr^3+^, Pb^2+^, and Fe^3+^ using bare AuNS. (d) Detection of Al^3+^, Cd^2+^, Zn^2+^, Hg^2+^, Ni^2+^, Cu^2+^, Cr^3+^, Pb^2+^, Fe^3+^, and Co^2+^ using silver nanospheres (AgNS_0.5_ and AgNS_1.0_). The subscripts 0.5 and 1.0 represent the nanoparticles concentration in terms of optical density. Sets 1–4 correspond to nanoparticles AgNS_0.5_, AgNS_1.0_, AuNS_0.5_, and AuNS_1.0_ respectively. The short dashed line shows the change in the sample compared to the control (marked by an arrow). The first centrifuge tube in each colorimetric panel represented as control for that panel.

A range of heavy metals, specifically As^3+^, Al^3+^, Cd^2+^, Zn^2+^, Hg^2+^, Ni^2+^, Cu^2+^, Cr^3+^, Pb^2+^, Fe^3+^, and Co^2+^, each with a concentration of 1 ppm, was examined. The isotropic CTAB-capped AgNS and AuNS were used to detect heavy metals without NaOH. Centrifuged CTAB-AgNS_0.5_ detected Hg^2+^ and Fe^3+^ based on changes in color and spectral absorbance compared to the control (Figure 5a).

Surprisingly, no metal detection was observed with centrifuged AgNS_1.0_ (Figure 5a). This might be due to the non-availability of binding sites for metals on the surface of silver nanoparticles, as CTAB is tightly bound to the isotropic nanoparticles as discussed earlier. However, as-prepared CTAB-AgNS_0.5_ and AgNS_1.0_ sensed Hg^2+^ and Cu^2+^ without NaOH (Figure 5b). The decrease in plasmonic intensity of as-prepared AgNS_0.5_ and AgNS_1.0_ in the presence of Hg^2+^ and Cu^2+^ leads to a colorimetric change (Figure 5b). This suggests the interaction of as-prepared CTAB-capped silver nanoparticles with Cu^2+^ and Hg^2+^, regardless of their concentration. The capping provides metal selectivity, whereas bare AgNS_1.0_ (without capping) showed unspecific metal detection such as Cd^2+^, Zn^2+^, Hg^2+^, Ni^2+^, Cu^2+^, Cr^3+^, Pb^2+^, Fe^3+^, and Co^2+^ (Figure 5d). However, in another study, only Hg^2+^ was detected with silver nanoparticles capped with poly(allylamine) hydrochloride but stabilized using CTAB [49]. Also, no prominent metal detection was achieved after adding NaOH to as-prepared CTAB-AgNS_0.5_ and AgNS_1.0_, confirming no interaction between metal and nanoparticles (Supporting Information File 1, Figure S2 and Figure S3). The as-prepared CTAB-AgNS contains 5612 µg/mL CTAB, which changes in the case of centrifuged CTAB-AgNS to 856 µg/mL. Despite less CTAB on the silver nanoparticle surface, they showed excellent sensing capability even at lower nanoparticle concentration. The addition of NaOH (5–50 µL) to centrifuged CTAB-AgNS led to no selectivity toward metal detection with centrifuged AgNS_1.0_, while aggregation of the particles was observed for AgNS_0.5_ starting from 20 µL of NaOH (Figure S4 and Figure S5, Supporting Information File 1). These results reveal that the as-prepared CTAB AgNS_0.5_ and AgNS_1.0_ and centrifuged AgNS_0.5_ selectively detect heavy metals in the absence of NaOH, while centrifuged AgNS_1.0_ showed interaction with multiple metals because of more particles with less CTAB than as-prepared particles. The bare/uncapped AgNS_0.5_ and AgNS_1.0_ detected various metals (Figure S10, Supporting Information File 1).

Gold nanoparticles with similar capping and size were considered to determine the role of surface capping in heavy metal detection. Interestingly, as-prepared and centrifuged CTAB-AuNS_0.5_ and AuNS_1.0_ showed no interaction with heavy metals without NaOH (Figure 5a,b). No metal ions were detected with as-prepared/centrifuged AuNS_0.5_ and AuNS_1.0_ in the absence and presence of NaOH (Figures S6–S9, Supporting Information File 1). However, bare AuNS detected various metals including Al^3+^, Ni^2+^, Cu^2+^, Cr^3+^, Pb^2+^, and Fe^3+^ (Figure 4c and Figure S10, Supporting Information File 1). The concentrations of CTAB in as-prepared and centrifuged AuNS are 30400 and 14460 µg/mL, respectively, which is far more than in silver nanoparticles. Silver nanoparticles interact with other metals because of their strong redox potential and the lower amount of CTAB [9]. The nanoparticle shape is also a criterion for designing nanoparticles as sensing probes. Therefore, we also assessed the potential of CTAB-capped gold nanorods (AuNR) as sensing probes for detecting heavy metals.

#### Anisotropic CTAB-capped gold nanorods

**Figure 6:**
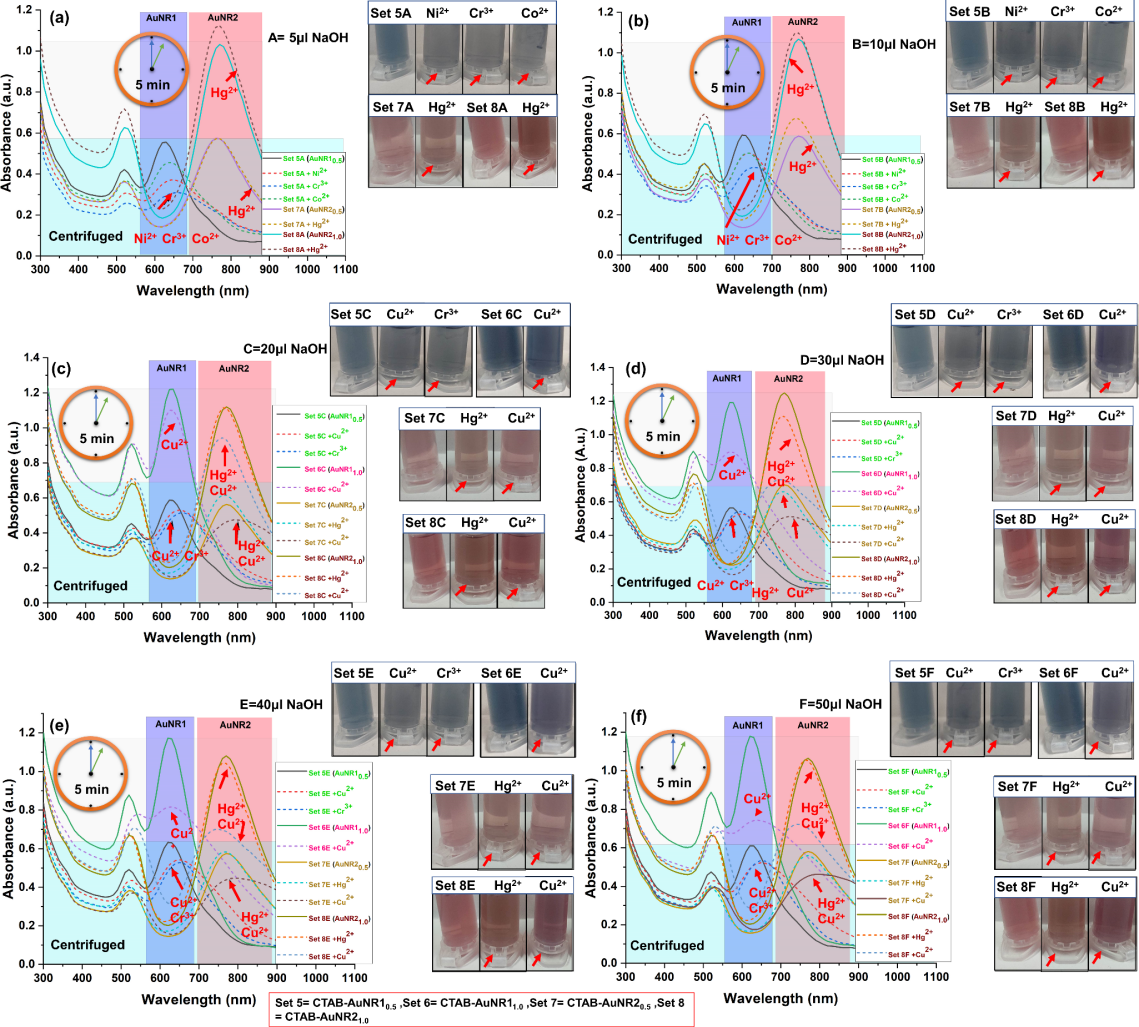
Spectrophotometric and colorimetric detection of heavy metals using centrifuged CTAB-AuNR1 and AuNR2 in the presence of NaOH. (a) AuNR1_0.5_ (set 5) and AuNR2_0.5/1.0_ (set 7 and set 8) detect Ni^2+^, Cr^3+^and Co^2+^, and Hg^2+^ in the presence of 5 μL NaOH (A). (b) AuNR1_0.5_ (set 5) and AuNR2_0.5/1.0_ (set 7 and set 8) detect Ni^2+^, Cr^3+^and Co^2+^, and Hg^2+^ in the presence of 5 μL NaOH (B); (c–f) AuNR1_0.5/1.0_ (set 5 and set 6) and AuNR2_0.5/1.0_ (set 7 and set 8) detect Cu^2+^, Cr^3+^, and Hg^2+^ in the presence of 20, 30, 40, and 50 µL of NaOH (C–F). The subscripts 0.5 and 1.0 represent the nanoparticle concentration in terms of optical density. Sets 5–8 correspond to nanoparticles AuNR1_0.5_, AuNR1_1.0_, AuNR2_0.5_ and AuNR2_1.0_, respectively. A, B, C, D, E, and F mean added NaOH volumes of 5, 10, 20, 30, 40, and 50 µL, respectively. The short-dashed line shows the change in the sample compared to the control (marked by an arrow). The first centrifuge tube in each colorimetric panel represented as control for that panel.

CTAB-AuNR1_0.5_ interacted with three metal ions, namely Ni^2+^, Cr^3+^, and Co^2+^, as evident through spectrophotometric and colorimetric differences compared to the control in the presence of 5–10 μL NaOH (Figure 6a,b).

The interaction of AuNR1_0.5_ with Hg^2+^ leads to a slight blueshift (no prominent color change), while redshifts were observed with other metals compared to the control (Supporting Information File 1, Figure S11). As discussed in previous sections, NaOH plays a significant role in decreasing the amount of CTAB on the nanoparticles, thus providing binding sites to the metal ions. The interaction with Hg^2+^ was also evident in as-prepared and centrifuged CTAB-AgNS. Placido et al. demonstrated Hg^2+^ detection using ʟ-cysteine-functionalized CTAB-AuNR [50]. However, multiple metals were detected, including Hg^2+^, without surface functionalization using NaOH (5–10 µL). After increasing the added amount of NaOH to 20–50 µL, centrifuged AuNR1_0.5_ selectively interacted with Cu^2+^ and Cr^3+^ with prominent color and spectral changes (Figure 6c–f). The metal selectivity switched upon increasing the volume of NaOH, confirming the significant role of NaOH in designing the nanoparticle-based sensor. Selective metal detection using centrifuged AuNR1_0.5_ was achieved among all metals in the presence of NaOH only (Supporting Information File 1, Figure S11). Centrifuged CTAB-AuNR1_0.5_ without NaOH showed no metal detection (Supporting Information File 1, Figure S11). Apart from the amount of added NaOH, the nanoparticle concentration in terms of OD also plays a significant role in the nanoparticle–metal interactions. Increasing the nanoparticle concentration, centrifuged AuNR1_1.0_ detected only copper (Cu^2+^) upon adding NaOH (30–50 µL) to the nanoparticles of 1 OD (Figure 6d–f). The colorimetric changes could be observed by the naked eye (Figure 6d–f). There were no visual differences in the centrifuged AuNR1_1.0_ plasmonic absorbance in the absence and presence of NaOH (5–20 µL) (Supporting Information File 1, Figure S12). Color or plasmon band changes can be attributed to nanoparticle–metal interactions [51]. Other researchers also explored the role of NaOH; they found that NaOH is a potent molecule that helps to remove CTAB from nanoparticles by reducing the CMC [33]. Indeed, the lower nanoparticle concentration (0.5 OD) provides high interaction due to the availability of fewer particles and less amount of CTAB on their surface compared to 1 OD with the same metal concentration (1 ppm). The concentrations of CTAB in as-prepared and centrifuged AuNR1 are 31020 and 13820 µg/mL, respectively. Thus, nanoparticle concentration also played a significant role in designing the detection system for heavy metals.

Centrifuged CTAB-AuNR2_0.5_ and AuNR2_1.0_ spectrophotometrically detected Hg^2+^ with 5–10 µL NaOH (Figure 6a,b). The results obtained with centrifuged CTAB-AuNR2_0.5_ and AuNR2_1.0_ were nearly the same as for centrifuged CTAB-AuNR1_0.5_ with Hg^2+^. However, in the case of centrifuged CTAB-AuNR2_0.5_, Hg^2+^ shows a prominent color change compared to centrifuged AuNR1_0.5_. The detection of Hg^2+^ is also obtained with centrifuged CTAB-AgNS_0.5_, but centrifuged CTAB-AuNS_0.5_ did not detect any metal ions. A subtle change in capping concentration on AuNR1 and AuNR2 was observed due to their length (Table 1). The change in concentration or size of nanorods ultimately affects the sensitivity towards metal ion detection. The metal selectivity was observed for all metal ions (Supporting Information File 1, Figure S13). With increasing nanoparticle concentration, centrifuged CTAB-AuNR2_1.0_ and AuNR2_0.5_ detected Hg^2+^ and Cu^2+^ with 20–50 µL NaOH (Figure 6c–f). The metal selectivity was changed upon increasing the NaOH volume and concentration of nanoparticles, as also observed for the shorter nanorods. The mechanism is similar to the interaction of metals with longer nanorods. However, the change in metal selectivity is due to the size of nanorods. In NaOH absence, no metal shows prominent interaction with centrifuged CTAB-AuNR2_1.0_ and AuNR2_0.5_ (Supporting Information File 1, Figure S13 and Figure S14). The absence of NaOH and no metal detection indicate a strong binding of CTAB on the nanoparticles’ surface. Therefore, it is certain that a minimum amount of NaOH is required to weaken the binding of CTAB without hampering the nanoparticles’ sensing abilities. Furthermore, the higher CTAB concentration in as-prepared samples was also evaluated to confirm metal selectivity. The concentrations of CTAB in as-prepared and centrifuged CTAB-AuNR2 are 30655 and 12315 µg/mL, respectively; the longer nanorods have a lower amount of bound CTAB. However, in the case of as-prepared samples, nearly the same amount of CTAB is present.

**Figure 7:**
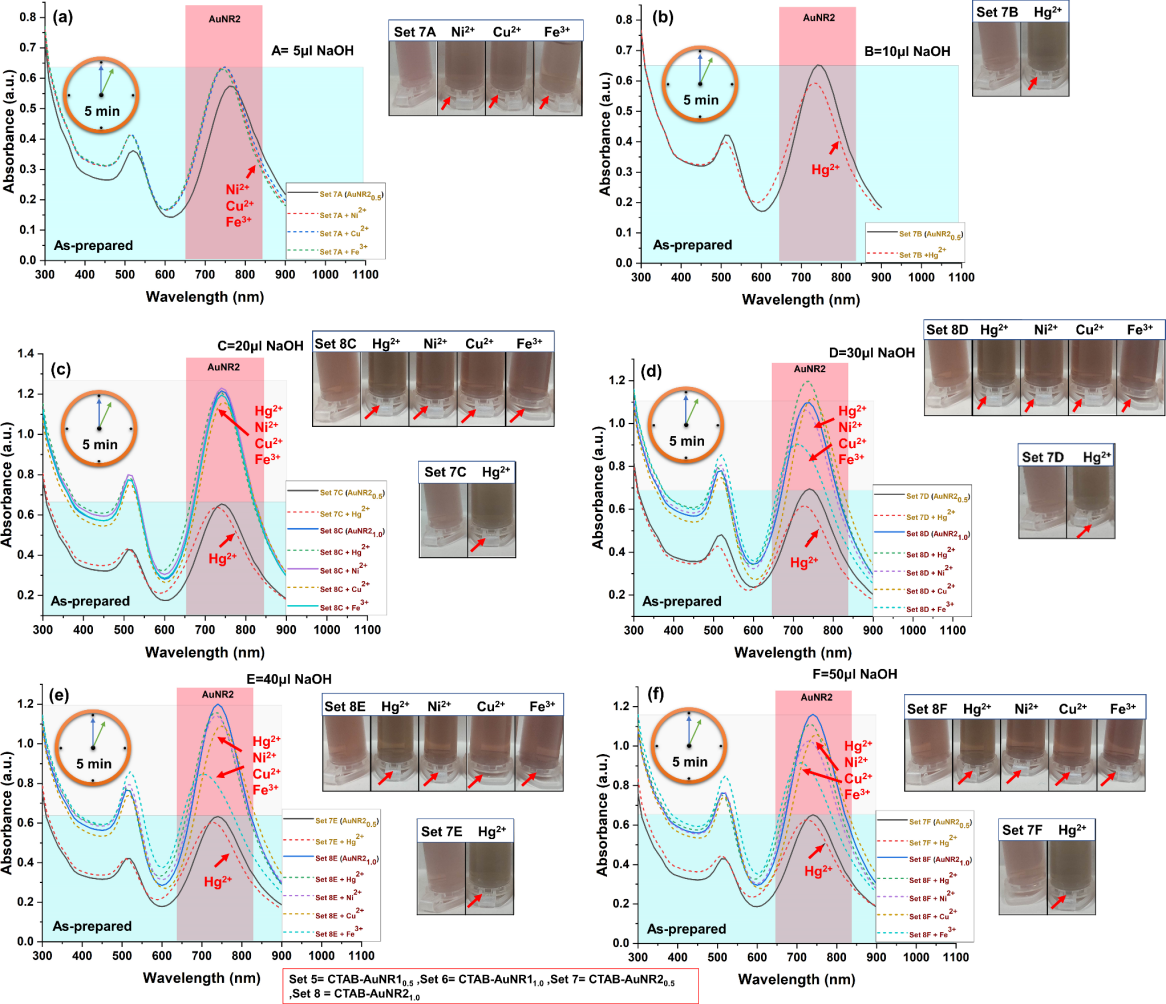
Spectrophotometric and colorimetric detection of heavy metals using as-prepared CTAB-AuNR2 in the presence of NaOH. (a) Only AuNR2_0.5_ (set 7) detects Ni^2+^, Cu^2+^, and Fe^3+^ in the presence of 5 μL NaOH (A). (b) AuNR2_0.5_ (set 7) detects Hg^2+^ in the presence of 10 µL NaOH (B). (c–f) AuNR2_0.5/1.0_ (set 7 and set 8) detect Hg^2+^, Ni^2+^, Cu^2+^, and Fe^3+^ in the presence of 20, 30, 40, 50 µL NaOH (C–F). The subscripts 0.5 and 1.0 represent the nanoparticle concentration in terms of optical density. Sets 5–8 correspond to nanoparticles AuNR1_0.5_, AuNR1_1.0_, AuNR2_0.5_ and AuNR2_1.0_, respectively. A, B, C, D, E, and F mean NaOH volumes of 5, 10, 20, 30, 40, and 50 µL, respectively, added to the nanoparticles. The short-dashed line shows the change in the sample compared to the control (marked by an arrow). The first centrifuge tube in each colorimetric panel represented as control for that panel.

In addition to this, the as-prepared CTAB-AuNR1 and CTAB-AuNR2 were also evaluated regarding heavy metal detection. Although the nanoparticle concentration has a big impact, washing nanoparticles is crucial in detecting heavy metals. As-prepared nanoparticles of the same concentration and size were evaluated to understand the impact of washing. As-prepared AuNR1 at 0.5 and 1 OD did not detect any metal through significant changes in color or plasmon resonance (Supporting Information File 1, Figure S15 and Figure S16). However, a slight blueshift of as-prepared CTAB-AuNR1_0.5_ was observed with Hg^2+^ only in the presence of various amounts (10–50 µL) of NaOH (Supporting Information File 1, Figure S15). This is evident by the blueshift of the plasmonic peak by 10 nm compared to the control samples. However, no prominent color change was observed (Supporting Information File 1, Figure S15). This is due to the excess CTAB on as-prepared AuNR1, which does not allow for interaction with metals. Only Hg^2+^ showed interaction, as observed in CTAB-AgNS, where the concentration of CTAB is much lower than in CTAB-AuNR1. Similarly, no heavy metal interaction is possible in the case of CTAB-AuNS due to tightly bound CTAB. However, in the case of CTAB-AuNR1, CTAB is tightly bound to the flat side and show an intermicellar gap at the curvature. The curvature allows for metal interaction only after adding a specific amount of NaOH. The nanoparticles (as-prepared AuNR1_0.5_ and AuNR1_1.0_) without NaOH showed no metal detection (Supporting Information File 1, Figure S15 and Figure S16). The robust nature of CTAB on nanoparticle surfaces is well known, thus resisting the interaction with target molecules without surface modification. In our case, NaOH was used to weaken CTAB micelles without hampering the physicochemical properties of the synthesized nanoparticles. This weakening of CTAB solves the persistent problem of surface modification or the use of linker molecules with CTAB-capped nanoparticles for sensing applications. In addition, the nanorod size might be crucial regarding nanorod–metal interactions. In a previous study conducted by Xu et al., Cu^2+^ was detected with cysteine-functionalized CTAB-AuNR (λ_max_ 800 nm), while Hg^2+^ was detected with cysteine-functionalized CTAB-AuNR (λ_max_ 650 nm) [50,52]. Both ʟ-cysteine-functionalized CTAB-AuNR detected different metals. Thus, it was confirmed that the size of the nanoparticles is a critical parameter influencing the physical and chemical properties of gold nanoparticles. The current study also evaluated size-dependent metal sensing using longer gold nanorods with the help of NaOH. Upon the addition of a specific volume of NaOH, the absorption band of CTAB-AuNR2 was blueshifted (λ_max_ 770 ± 5 nm to 740 ± 5 nm). The shifted peak of CTAB-AuNR2 was used as a control for further heavy metal sensing experiments because no aggregation was observed. As-prepared CTAB-AuNR2_0.5_ interacted only with Hg^2+^ with a prominent blueshift of the plasmonic peak after addition of 10–50 µL of NaOH (Figure 7b–f). The present study demonstrated that merely altering the parameters of nanorods is sufficient for the detection of heavy metals, eliminating the need for any additional linker or buffer.

As-prepared AuNR2_0.5_ interacted with Ni^2+^, Cu^2+^, and Fe^3+^ metal ions in the presence of 5 μL NaOH (Figure 7a). However, no significant change in nanoparticles was observed in the absence of NaOH compared to the control (Supporting Information File 1, Figure S17). As-prepared CTAB-AuNR2_1.0_ showed interaction with Hg^2+^, Ni^2+^, Cu^2+^, and Fe^3+^ in the presence of 20–50 µL of NaOH with a prominent blueshift of the absorption band (Figure 7c–f). The metal selectivity changed in the case of as-prepared CTAB-AuNR2_1.0_ compared to centrifuged AuNR2_1.0_ and AuNR1, which might be due to the size of nanorods or shifting of the control sample peak upon the addition of NaOH. The as-prepared AuNR2_1.0_ showed no plasmonic or colorimetric change with metal ions in the absence and presence of 5–20 µL NaOH (Figure 7a–c and Supporting Information File 1, Figure S18). The color and plasmon change confirmed the metal–nanoparticle interactions. Gold nanorods of two sizes (AuNR1 and AuNR2) detected different metals, suggesting that size, concentration, and centrifuged/as-prepared nanorods are critical parameters for designing a sensing probe for heavy metals. Shape and metal (with the same capping) were also evaluated for their role in heavy metal detection. Table 2 summarizes various studies on detecting heavy metals using CTAB-capped metal nanoparticles via linkers or buffers. Table 2 includes detected metals using as-prepared/centrifuged CTAB-capped AgNS, AuNS, AuNR1, and AuNR2.

**Table 2:** Studies related to CTAB-capped gold and silver nanoparticles for the detection of heavy metals.

CTAB-capped nanoparticles	Ligand/linker	Buffer	Metal detected	Reference

AgNP	poly(ʟ-lysine)	—	Hg^2+^	[9]
AuNS (λ_max_ 530 nm)	sodium thiosulfate	—	Pb^2+^	[24]
AuNR (λ_max_ 650 nm)	ʟ-cysteine	—	Hg^2+^	[50]
AuNR (λ_max_ 801 nm)	cysteine	—	Cu^2+^	[52]
AuNR (λ_max_ 655 nm)	*N*-alkylaminopyrazole ligand, 1-[2-(octylamino)ethyl]-3,5-diphenylpyrazole	—	Hg^2+^	[53]
AuNR (λ_max_ 732 nm)	TPDT-silicate	—	Hg^2+^	[54]
AuNR (λ_max_ 625 nm)	Na_2_S_2_O_3_	NH_3_/NH_4_Cl buffers (pH 10.4)	Cu^2+^	[55]
AuNR	—	hydrogen bromide buffer solution	Cu^2+^	[56]
AuNR (λ_max_ 700 nm)	dithiothreitol (DTT)	—	As^3+^	[57]
AuNR	ʟ-arginine	—	Hg^2+^	[58]
AgNS (λ_max_ 410 nm)	—	—	Hg^2+^, Cu^2+^, and Fe^3+^	this work^a^
AuNS (λ_max_ 525 nm)	—	—	—	this work^a^
AuNR1 (λ_max_ 630 nm)	—	—	Cr^3+^, Co^2+^, Ni^2+^, and Cu^2+^	
AuNR2 (λ_max_ 770 nm)	—	—	Hg^2+^, Ni^2+^, Fe^3+^, and Cu^2+^	

^a^The metal detection depends on size, shape (isotropic and anisotropic), concentration (0.5 and 1 OD), and the nature (centrifuged or as-prepared) of the nanoparticles and the volume of added NaOH.

#### Possible mechanism of heavy metal detection

Previous studies have established that CTAB prevents interactions with ligands such as metal ions. Consequently, alternative linkers have typically facilitated metal ion detection using CTAB-capped nanoparticles. However, this study has developed a system capable of detecting metal ions without such linkers. The detection mechanism in this study is influenced by factors such as the amount of NaOH, nanoparticle shape, and nanoparticle concentration. As previously reported, CTAB-capped silver nanospheres detected Cu^2+^ and Hg^2+^, attributed to the interaction between the metal ions and the nanoparticles. The underlying mechanism is based on the higher affinity of Hg^2+^ towards silver, with a binding energy of 1.78 eV [9]. This strong affinity allows Hg^2+^ ions to penetrate the CTAB bilayer, particularly in AgNS, where the amount of bound CTAB is lower [9]. Additionally, Cu^2+^ ions undergo a galvanic replacement reaction with silver, facilitated by their high redox potential, enabling Cu–Ag interaction in low-bound CTAB nanoparticles [59]. In contrast, gold nanospheres did not exhibit any metal interactions in the presence of NaOH, which is attributed to the tightly bound CTAB on AuNS surfaces. However, CTAB-AuNS was reported to detect Pb^2+^ in the presence of sodium thiosulfate due to the thiol group’s higher affinity for metal ions [24]. Furthermore, the study found that AuNR1 and AuNR2 detected metals only in the presence of NaOH, possibly due to the removal of CTAB from the nanorod curvature, which is known to contain less CTAB. Centrifuged AuNR1_0.5_ nanorods detected Cr^3+^, Co^2+^, Ni^2+^, and Cu^2+^ with NaOH volumes of 5–10 μL. Upon increasing the NaOH volume, only Cr^3+^ and Cu^2+^ were detected with AuNR1_0.5_ and AuNR1_1.0_ nanorods. This suggests that NaOH plays a crucial role in facilitating nanorod–metal interactions through surface CTAB dissolution. Similar to the interaction between copper and silver, copper and gold can undergo galvanic replacement. Gold ions in solution can replace copper atoms in nanoparticles, forming bimetallic structures [60]. It is assumed that Cr^3+^ and Cu^2+^ ions interacted directly with CTAB and adsorbed onto the nanorod surface. Previous studies have shown that CTAB-AuNR in hydrogen bromide-buffered solution and coated with Na_2_S_2_O_3_ in NH_3_/NH_4_Cl buffer selectively detect Cu^2+^ [55–56]. Additionally, ʟ-cysteine-coated gold nanorods have been reported to detect Cu^2+^ [52]. These findings suggest that the interaction between CTAB and Cu^2+^ is independent of the ligand or buffer, as Cu^2+^ was detected across different conditions. In this study, NaOH was employed to weaken CTAB on nanorods, potentially allowing for the detection of multiple metals, which was not observed in previous studies (Table 2). Using longer nanorods (AuNR2), different metals such as Hg^2+^, Cu^2+^, Ni^2+^, and Fe^3+^ were detected. The underlying mechanism is similar to that of AuNR1, where the addition of NaOH leads to weak binding of CTAB on the nanorod surface. This allowed Hg^2+^ to interact with Au due to its high affinity, similar to its interaction with Ag. Previous reports have shown that ʟ-cysteine-, TPDT-silicate-, and ʟ-arginine-coated AuNR can detect Hg^2+^ despite different ligand conjugations [50,54,58]. These findings suggest that metal–ion interactions with CTAB-capped nanoparticles occur through ionic interactions with the surface capping. Once the surface capping is weakened, multiple metals, rather than a single metal, can be detected. This approach enhances the sensing ability regarding various metal ions without the need for additional linkers, ligands, or buffers. The current study demonstrates superior performance compared to traditional methods and establishes a novel multimetal detection system using CTAB-capped nanoparticles, which has not been reported before.

#### LOD and LOQ quantification

This study confirmed the detection of heavy metals using CTAB-capped metal nanoparticles by changing the parameters such as size, shape, as-prepared/centrifuged, and concentration of the nanoparticles, as well as the volume of added NaOH solution. However, the detected metals were at 1 ppm concentration. Therefore, it is impossible to calculate the minimum detection limit because the metal detection happened at a minimum threshold of 1 ppm. Despite that, this study allows other researchers to design nanosensors without surface modification or the use of linker molecules for multimetal detection. However, pristine isotropic gold and silver nanoparticles (without capping) detect multiple metals at 1 ppm concentration, including chromium (Supporting Information File 1, Figure S10). Furthermore, the LOD of Cr^3+^ was calculated using bare AuNS using a linear plot in the range of 100–400 ppb (Supporting Information File 1, Figure S19a,b). The LOD and LOQ of Cr^3+^ using pristine gold nanoparticles were 469.34 and 1422.25 ppb, respectively. Even though CTAB-capped nanoparticles do not go beyond 1 ppm, they are selective for metal detection, which is not the case for pristine gold nanospheres. Thus, CTAB-capped metal nanoparticles can be tailored for heavy metal detection without using linkers or buffers by modifying parameters such as size, shape, concentration, as-prepared/centrifuged, and the volume of NaOH solution.

#### Catalytic efficiency of CTAB-capped nanoparticles

**Figure 8:**
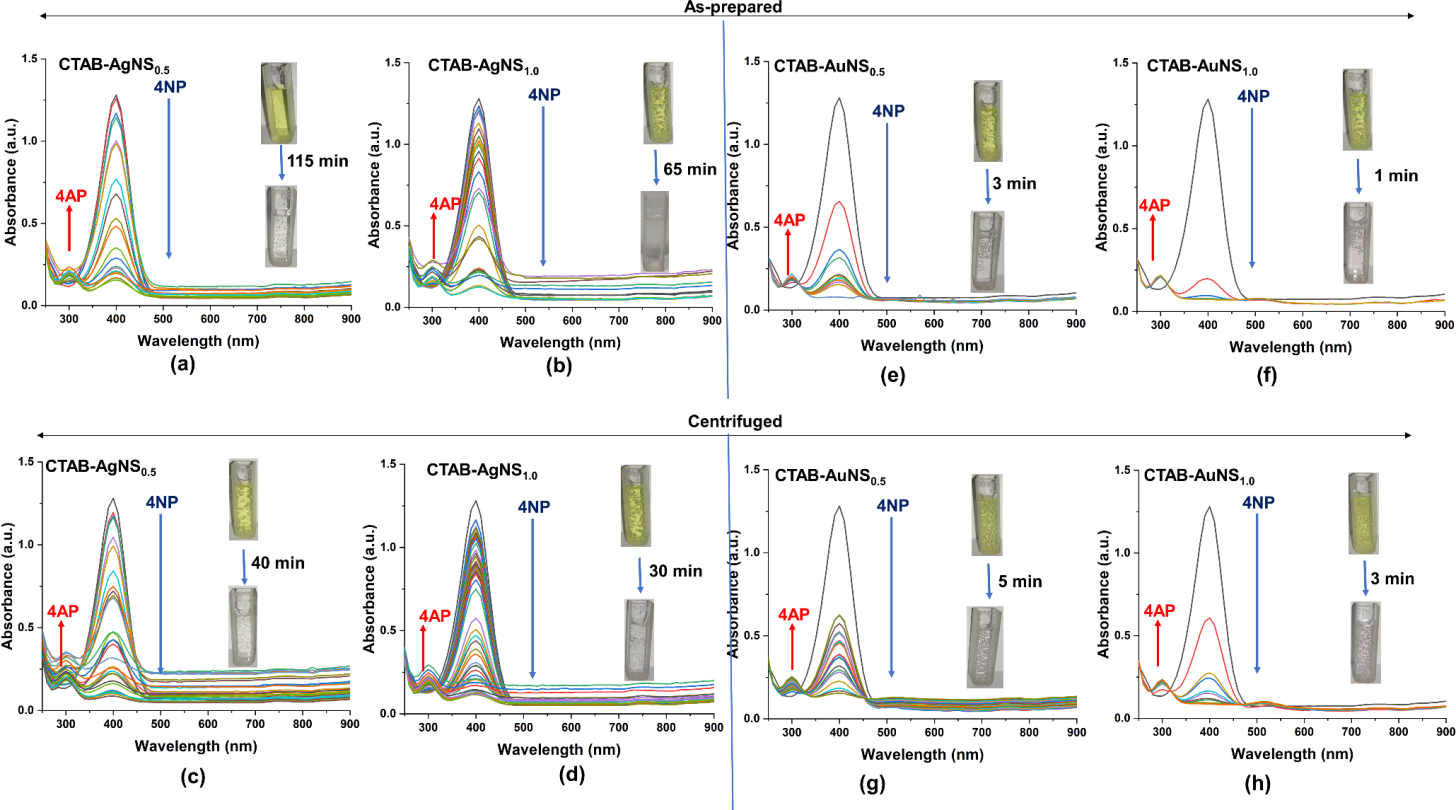
Degradation of 4 nitrophenol (4-NP) to 4 aminophenol (4-AP) using CTAB-AgNS and AuNS. (a) As-prepared AgNS_0.5_, (b) as-prepared AgNS_1.0_, (c) centrifuged AgNS_0.5_, (d) centrifuged AgNS_1.0_, (e) as-prepared AuNS_0.5_, (f) as-prepared AuNS_1.0_, (g) centrifuged AuNS_0.5_, and (h) centrifuged AuNS_1.0_. The inset color shows a change in the degradation of 4-NP to 4-AP. The subscripts 0.5 and 1.0 represent the nanoparticle concentration in terms of optical density.

The catalytic efficiency of CTAB-capped metal nanoparticles was investigated using 4-nitrophenol, a commonly employed substrate for assessing the catalytic efficiency of nanoparticles, as a substrate. Previous studies on CTAB-capped nanoparticles have suggested their role as a catalytic agent for the conversion of 4-NP to 4-AP in the presence of NaBH_4_ [27]. All catalysis experiments were conducted in situ by performing the reduction reaction in a standard 1 cm path length quartz cuvette and measuring the spectral changes as the reaction progressed. The experimental setup involved an aqueous solution of 0.1 mM 4-NP and 0.1 M NaBH_4_, followed by the addition of CTAB-capped nanoparticles. Immediately after the addition of NaBH_4_, a color change from light yellow to yellow-green was observed; simultaneously, the spectral absorption changed from 317 to 400 nm due to the formation of 4-nitrophenolate ions in the reaction medium [61]. The reduction of 4-nitrophenolate ions to 4-aminophenol was initiated only upon the addition of nanoparticles as a catalyst, and reaction kinetics were monitored using a UV–visible spectrophotometer. The visible change from yellow to a colorless solution corresponded to a decrease in the peak intensity at 400 nm and the formation of a minor peak around 300 nm due to the formation of 4-aminophenol. The catalytic conversion of 4-nitrophenol by as-prepared CTAB-AgNS_0.5_ and CTAB-AgNS_1.0_ was completed in 115 and 65 min with catalytic efficiencies of 87.82% and 89.14%, respectively, (Figure 8a,b).

**Figure 9:**
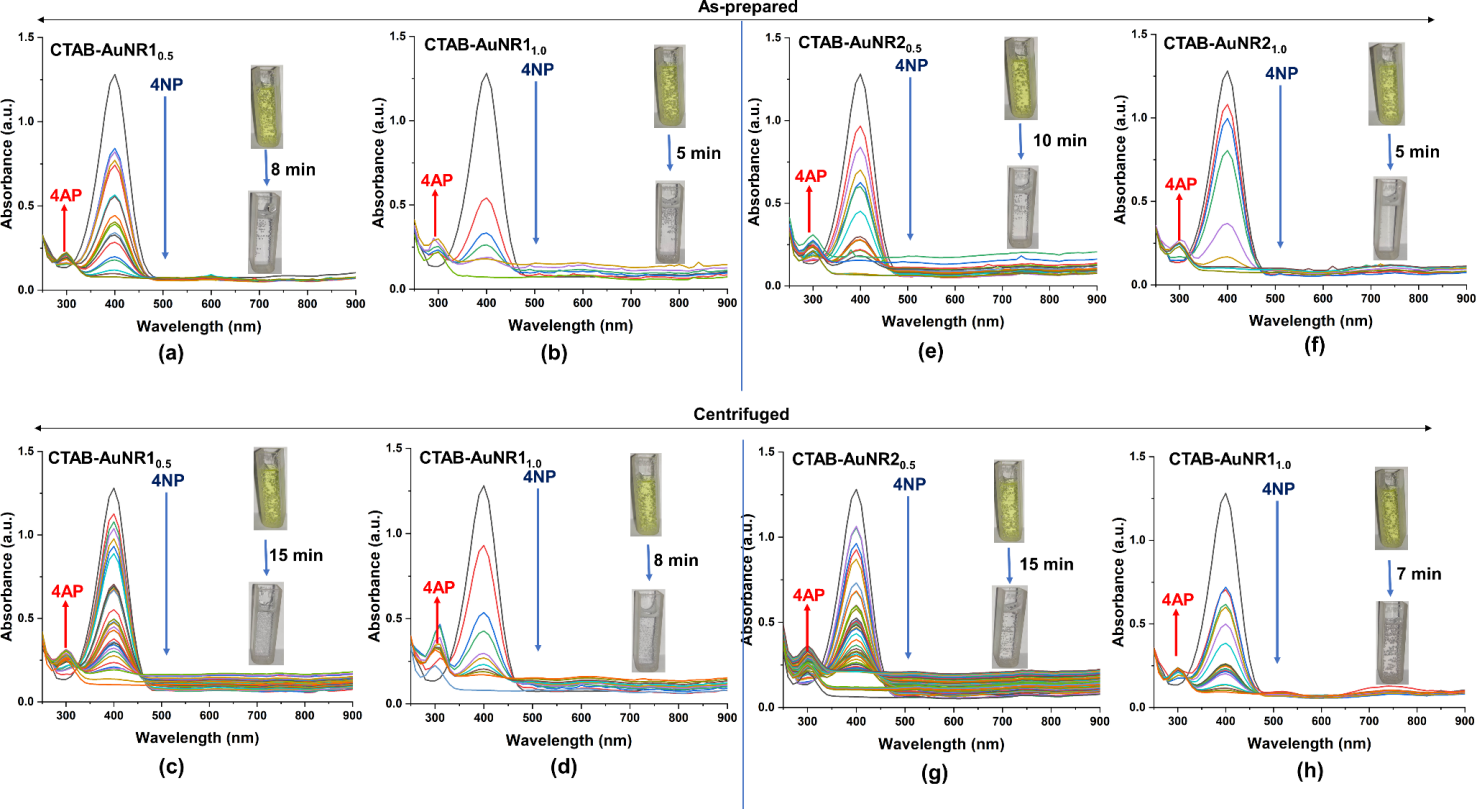
Degradation of 4 nitrophenol (4-NP) to 4 aminophenol (4-AP) using CTAB-AuNR1 and AuNR2. (a) As-prepared AuNR1_0.5_, (b) as-prepared AuNR1_1.0_, (c) centrifuged AuNR1_0.5_, (d) centrifuged AuNR1_1.0_, (e) as-prepared AuNR2_0.5_, (f) as-prepared AuNR2_1.0_, (g) centrifuged AuNR2_0.5_, and (h) centrifuged AuNR2_1.0_. The inset shows the change in degradation of 4-NP to 4-AP. The subscripts 0.5 and 1.0 represent the nanoparticles concentration in terms of optical density.

Using centrifuged CTAB-AgNS_0.5_ and CTAB-AgNS_1.0_, the degradation times were 40 and 30 min with catalytic efficiencies of 93.13% and 90.78%, respectively (Figure 8c,d). Shaikh et al. showed the degradation of 4-NP using ginger extract-stabilized CTAB-capped silver nanospheres [62]. However, the current study observed the degradation of 4-NP based on nanoparticle concentration and preparation conditions without using any linker. Centrifuged particles are more efficient compared to as-prepared nanoparticles. In comparison, CTAB and pristine AgNPs (1 OD) degraded 4-NP within 60 and 40 min, respectively (Supporting Information File 1, Figure S20). CTAB-AgNPs degrade 4-NP faster because of a synergistic effect. Furthermore, the impact of the metal with the same capping was also evaluated. Using as-prepared CTAB-AuNS_0.5_ and CTAB-AuNS_1.0_, the degradation of 4-NP was achieved in 3 and 1 min with catalytic efficiencies of 93.91% and 94.37%, respectively (Figure 8e,f). The present findings corroborate those reported by Satapathy and colleagues. The authors concluded that the degradation of 4-NP highly depends on the nanoparticle concentration, which is also observed in our study [61]. Compared to the as-prepared nanoparticles, centrifuged CTAB-AuNS_0.5_ and AuNS_1.0_ took 5 and 3 min with catalytic efficiencies of 87.97% and 93.20%, respectively, for the degradation of 4-NP (Figure 8g,h). The time required for the degradation of 4-NP using centrifuged gold nanospheres is higher than that using as-prepared AuNS. This might be due to the lower amount of CTAB in centrifuged AuNS. The degradation times of 4-NP using CTAB and bare/uncapped AuNS were 60 and 5 min, respectively (Supporting Information File 1, Figure S20). This confirmed that gold nanoparticles have catalytic activity against 4-NP, enhanced upon capping with CTAB. However, it was reported that the shape of nanoparticles also influences their catalytic property in the degradation of 4-NP [63]. Figure 9a,b shows the decrease in optical absorption of 4-NP (λ_max_ 400 nm) using as-prepared CTAB-AuNR1_0.5_ and AuNR1_1.0_ as reaction catalysts. The Figure inset represents a color change from yellow to colorless obtained within 8 and 5 min with catalytic efficiencies of 93.83% and 94.37%, respectively, after the addition of as-prepared CTAB-AuNR1_0.5_ and AuNR1_1.0_.

A similar trend was observed in the case of centrifuged AuNR1, where a high nanoparticle concentration (AuNR1_1.0_) required less time than a lower concentration (AuNR1_0.5_) (Figure 9c,d). The degradation times of 4-NP using centrifuged AuNR1_0.5_ and AuNR1_1.0_ were 8 and 5 min with catalytic efficiencies of 91.95% and 93.83%, respectively. This confirmed that as-prepared AuNR1 showed higher efficiency for the degradation of 4-NP compared to centrifuged AuNR1. This might be due to excess CTAB in as-prepared AuNR1, which is also the case with CTAB-AuNS. CTAB, without nanoparticles, can reduce 4-NP to 4-AP (Supporting Information File 1, Figure S20a). However, the degradation time was much higher than that of the CTAB-capped nanoparticles, as observed in the case of CTAB-AuNR1. We also analyzed the role of nanoparticle size in the degradation of 4-NP. Therefore, the potential of longer CTAB-AuNR was also evaluated. As-prepared CTAB-AuNR2_0.5_ and AuNR2_1.0_ showed degradation times of 10 and 5 min with catalytic efficiencies of 94.76% and 93.83%, respectively (Figure 9e,f). Centrifuged CTAB-AuNR2_0.5_ and AuNR2_1.0_ degraded 4-NP within 15 and 7 min with catalytic efficiencies of 95.01% and 92.74%, respectively, similar to the case for CTAB-AuNR1 (Figure 9g,h). A slight change in degradation time was observed due to the size difference of the gold nanorods. A trend reversal in CTAB-AgNS was observed, where centrifuged AgNS showed minimal degradation time compared to as-prepared. The 4-NP degradation time of centrifuged CTAB-AgNS was comparable to that of bare AgNS (Supporting Information File 1, Figure S20). This study suggests that the degradation of 4-NP using nanoparticles highly depends on nanoparticle concentration, size, shape, and preparation. Table 3 summarizes various studies performed regarding the degradation of 4-nitrophenol using nanoparticle-based systems.

**Table 3:** Studies related to nanoparticles for the degradation of 4-nitrophenol.

System	Shape	Degradation time (min)	Reference

CTAB-AuNP	sphere	1	[61]
CTAB-AgNP and bare AgNP (in extract)	sphere	3	[62]
Silica coated CTAB-AuNR	rod	5–6	[64]
*Cochliobolus geniculatus* coated ZnO nanoparticles	quasi-spherical	30	[65]
*B. amyloliquefaciens* capped silver nanoparticles	sphere	15	[66]
Jatropha leaf extract capped iron nanoparticles	irregular shaped	60	[67]
polyvinyl alcohol/silver nanoparticle film	sphere	25	[68]
CTAB AgNS	sphere	30	this work^a^
CTAB-AuNS	sphere	1	
CTAB-AuNR1	rod	5	
CTAB-AuNR2	rod	5	

^a^The obtained degradation time depends on nanoparticle concentration, size, shape and preparation of the nanoparticles.

#### Possible mechanism of 4-NP degradation

The catalytic degradation of 4-NP was achieved using CTAB-capped nanoparticles as nanocatalysts, where CTAB-AuNS is highly efficient compared to CTAB-AgNS, CTAB-AuNR1, and CTAB-AuNR2. The underlying mechanism behind 4-NP degradation using CTAB-capped gold and silver nanoparticles involves a complex series of surface-mediated reactions. Initially, 4-NP in its ionic form (4-nitrophenolate) penetrates the CTAB bilayer and adsorbs onto the metal nanoparticle surface, facilitated by electrostatic interactions [69–70]. Concurrently, borohydride ions (BH^4−^) from NaBH_4_ also adsorb on the nanoparticle surface. The metal nanoparticle acts as an electron relay, accepting electrons from BH^4−^ and transferring them to the adsorbed 4-nitrophenolate [69]. This electron transfer initiates a stepwise reduction of the nitro group, proceeding through nitroso and hydroxylamine intermediates before finally yielding 4-aminophenol (4-AP).

The reduction follows the pathway: 4-NO_2_-phenolate → 4-NO-phenolate → 4-NHOH-phenolate → 4-NH_2_-phenolate. Once formed, 4-AP desorbs from the nanoparticle surface, allowing the catalytic cycle to continue [69]. The efficiency of this process is influenced by nanoparticle properties such as surface area, size, and shape, with smaller particles generally exhibiting higher catalytic activity due to their increased surface-to-volume ratio.

## Conclusion

This study developed a flexible nanosensor without linkers, using cetyltrimethylammonium bromide as capping agent on gold and silver nanoparticles. The nanosensor was designed to rapidly detect heavy metal ions and degrade organic pollutants like 4-nitrophenol. CTAB-AgNS, CTAB-AuNS, CTAB-AuNR1, and CTAB-AuNR2 nanoparticles were characterized regarding size, shape, surface charge, functional group interactions, and crystalline structure. Stability tests with NaCl and NaOH showed that CTAB-AgNS exhibited decreased plasmonic intensity and aggregation, while CTAB-AuNS and CTAB-AuNR1 demonstrated higher stability than CTAB-AuNR2. CTAB-AuNS efficiently degraded 4-nitrophenol (94.37% within 1 min), while CTAB-AuNR1 and AuNR2 degraded it within 5 min with an efficiency of 94.37% and 93.83%, respectively. The efficiency of the sensing probes depended on factors like nanoparticle concentration, preparation method (as-prepared/centrifuged), and NaOH addition. As-prepared CTAB-AgNS detected Cu^2+^ and Hg^2+^ ions at specific concentrations, whereas CTAB-AuNR1 and AuNR2 detected multiple metals under particular conditions. CTAB-AuNS showed no metal ion detection capability. These results highlight the importance of size, shape, concentration, and preparation method of the nanoparticles in sensing and catalytic applications. The sensor achieved metal ion detection at 1 ppm without linkers or buffer solvents and detected multiple metal ions based on reaction parameters. Future research will enhance sensitivity towards metal detection using linker-free CTAB-capped metal nanoparticles combined with advanced strategies. This study demonstrates the potential of linker-free CTAB-capped metal nanoparticles for simultaneous pollutant detection and degradation, offering promising environmental remediation and sensing applications.

## Supporting Information

The file contains twenty figures. Figure S1 is an HPLC chromatogram showing peak retention time for CTAB and CTAB-capped nanoparticles. Figures S2–S9 are spectrophotometric and colorimetric visualizations of metal detection using isotropic CTAB-capped nanoparticles. Figure S10 depicts the spectrophotometric and colorimetric detection of metal ions using bare gold and silver nanoparticles. Figures S11–S18 show the detection of metal ions using anisotropic nanoparticles of gold (i.e., AuNR1 and AuNR2). Figures S19 and S20 present the quantification of Cr^3+^ using bare AuNS and the catalytic degradation of 4-NP with CTAB, bare AuNS and bare AgNS, respectively.

File 1 Additional figures.

## Data Availability

The data that supports the findings of this study is available from the corresponding author upon reasonable request.
