# Supplementary material for: Nanoarchitectonics with cetrimonium bromide on metal nanoparticles for linker-free detection of toxic metal ions and catalytic degradation of 4-nitrophenol
Source: Beilstein J Nanotechnol. 2024 Nov 4;15:1312–32. doi: 10.3762/bjnano.15.106 (PMC11552433; doi:10.3762/bjnano.15.106)
Supplement: File 1 — Additional figures. [file Beilstein_J_Nanotechnol-15-1312-s001.pdf]

BEILSTEIN JOURNAL OF NANOTECHNOLOGY

Supporting Information

for

Nanoarchitectonics with cetrimonium bromide on metal nanoparticles for linker-free detection of toxic metal ions and catalytic degradation of 4-nitrophenol

Akash Kumar and Raja Gopal Rayavarapu

Beilstein J. Nanotechnol. **2024,** *15,* 1312–1332. doi:10.3762/bjnano.15.106

Additional figures

License and Terms: This is a supporting information file under the terms of the Creative Commons Attribution License (https://creativecommons.org/licenses/by/4.0). Please note that the reuse, redistribution and reproduction in particular requires that the author(s) and source are credited and that individual graphics may be subject to special legal provisions.
The license is subject to the *Beilstein Journal of Nanotechnology* terms and conditions: (https://www.beilstein-journals.org/bjnano/terms)

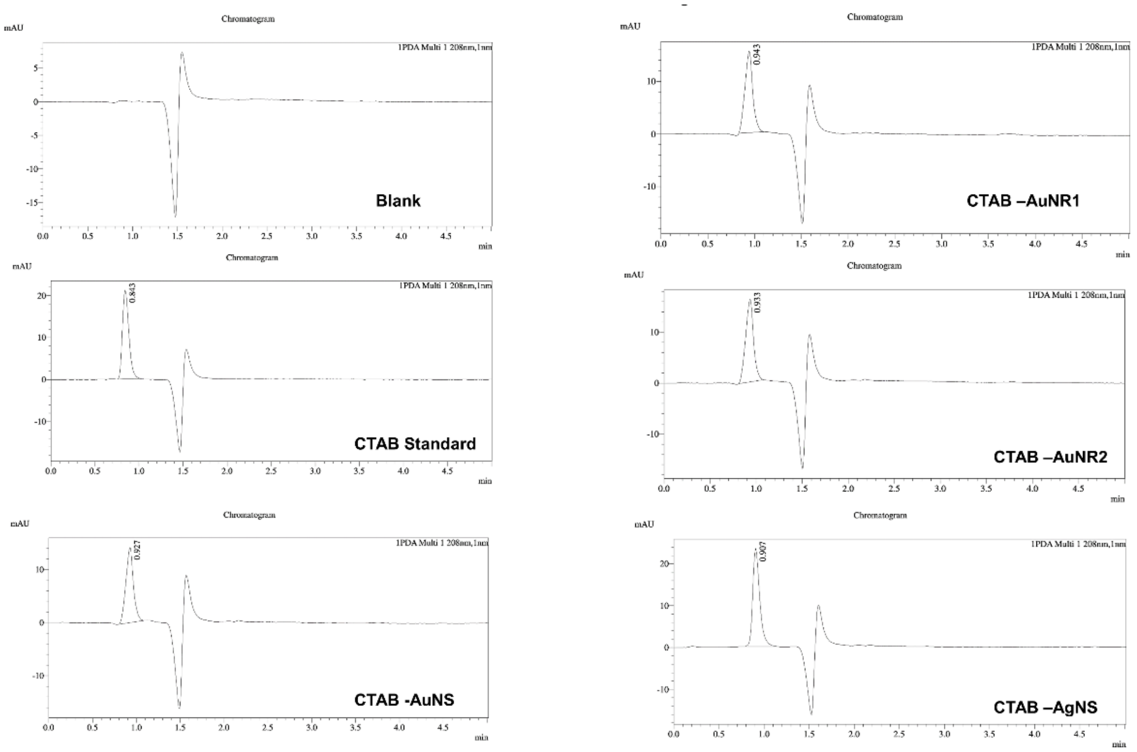

Figure S1: HPLC chromatogram of parent CTAB along with CTAB capped AuNS, AgNS, AuNR1 and AuNR2.

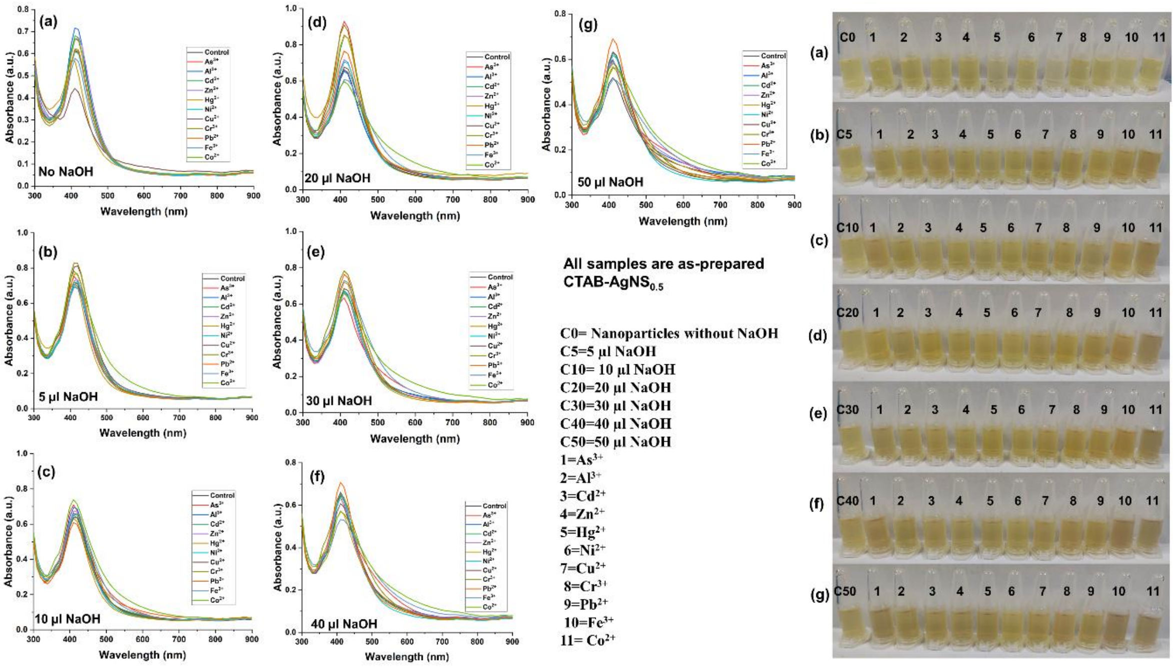

Figure S2: Spectrophotometric and colorimetric detection of heavy metal ions using as-prepared CTAB-AgNS at 0.5 OD in the absence (a) and presence (b-g) of NaOH (5-50 µL).

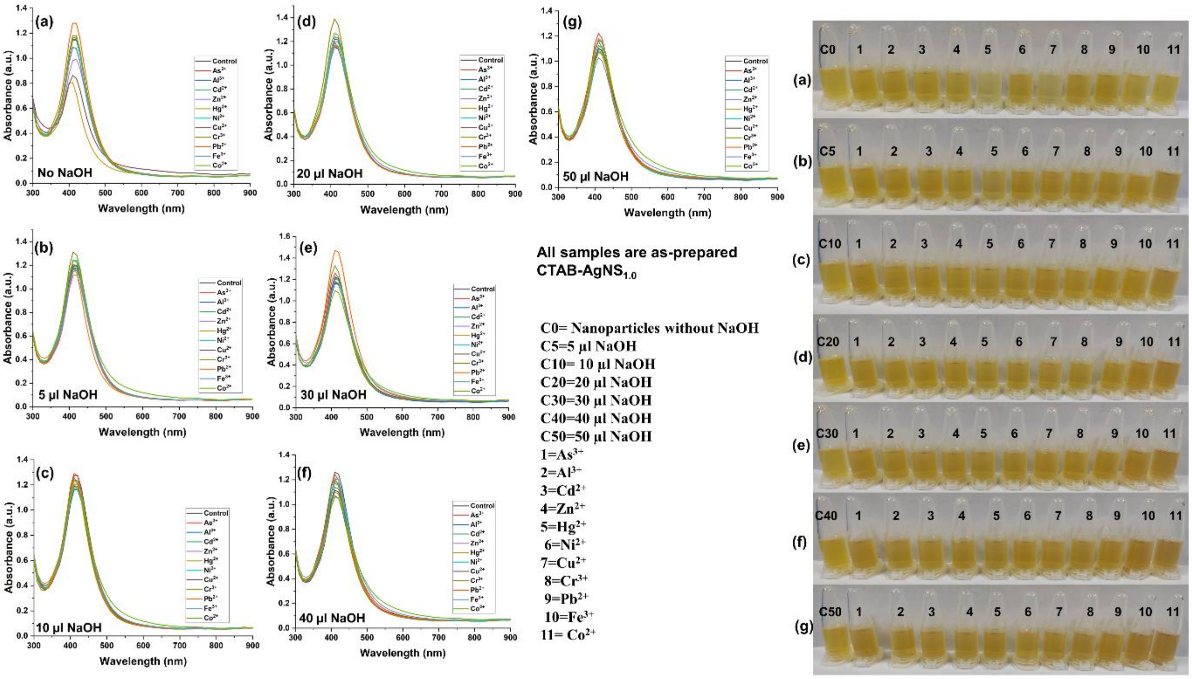

Figure S3: Spectrophotometric and colorimetric detection of heavy metal ions using as-prepared CTAB-AgNS at 1 OD in the absence (a) and presence (b-g) of NaOH (5-50 µL).

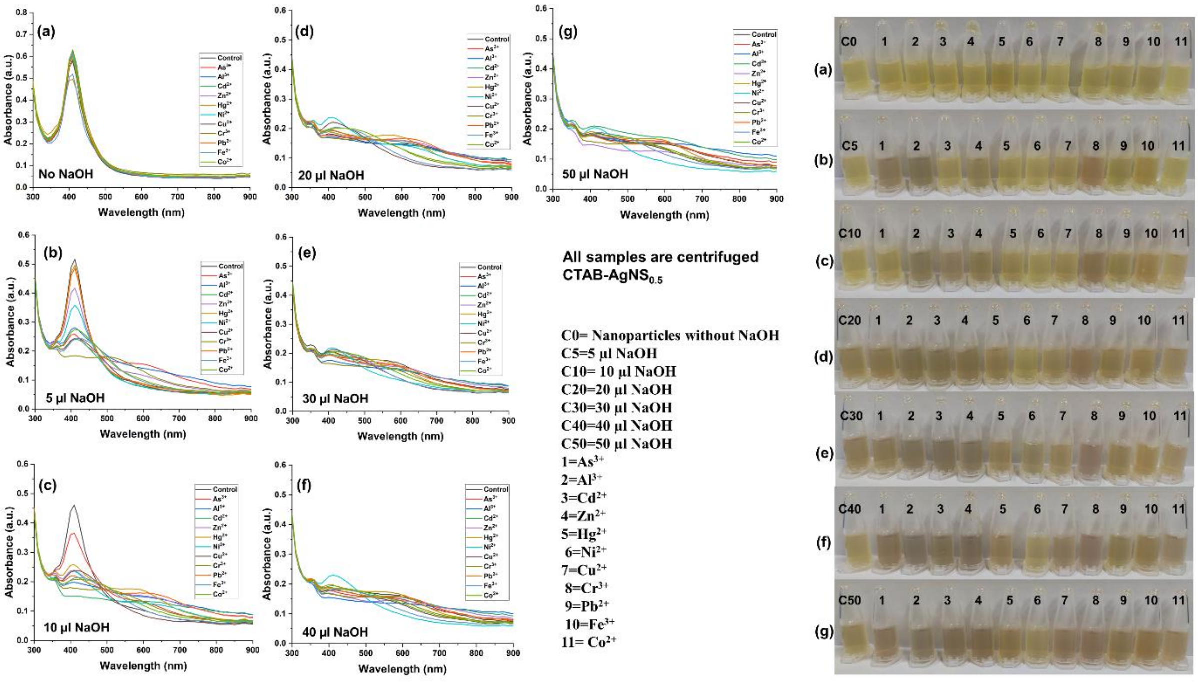

Figure S4: Spectrophotometric and colorimetric detection of heavy metal ions using centrifuged CTAB-AgNS at 0.5 OD in the absence (a) and presence (b-g) of NaOH (5-50 µL).

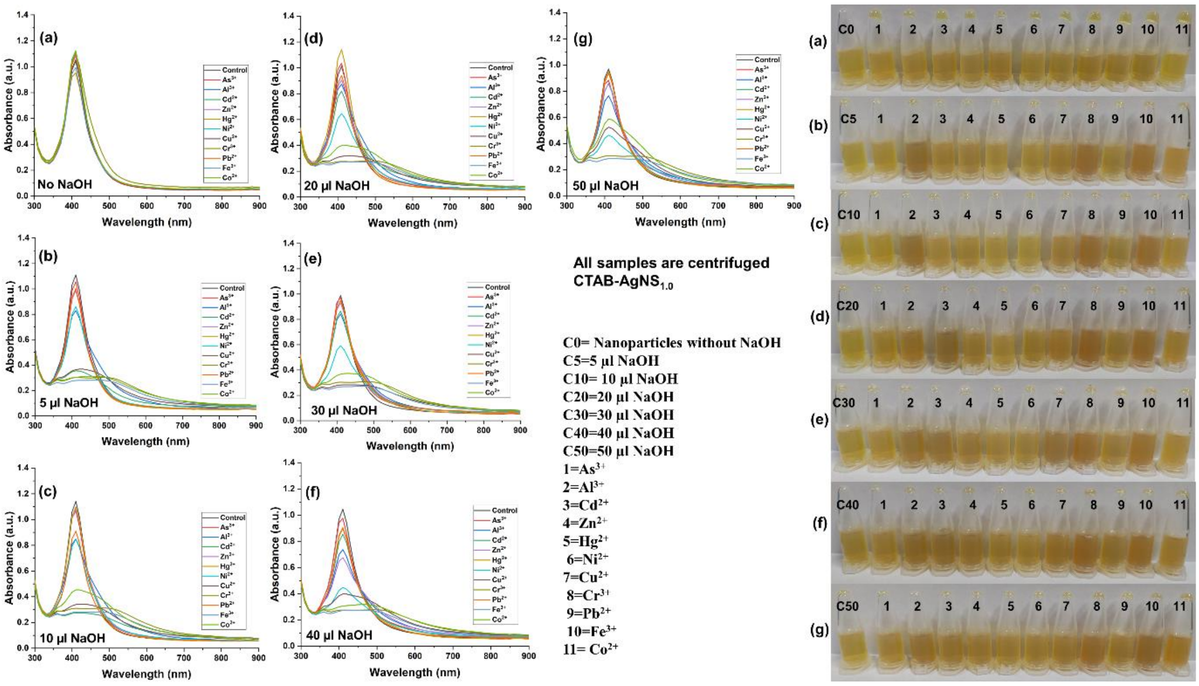

Figure S5: Spectrophotometric and colorimetric detection of heavy metal ions using centrifuged CTAB-AgNS at 1 OD in the absence (a) and presence (b-g) of NaOH (5-50 µL).

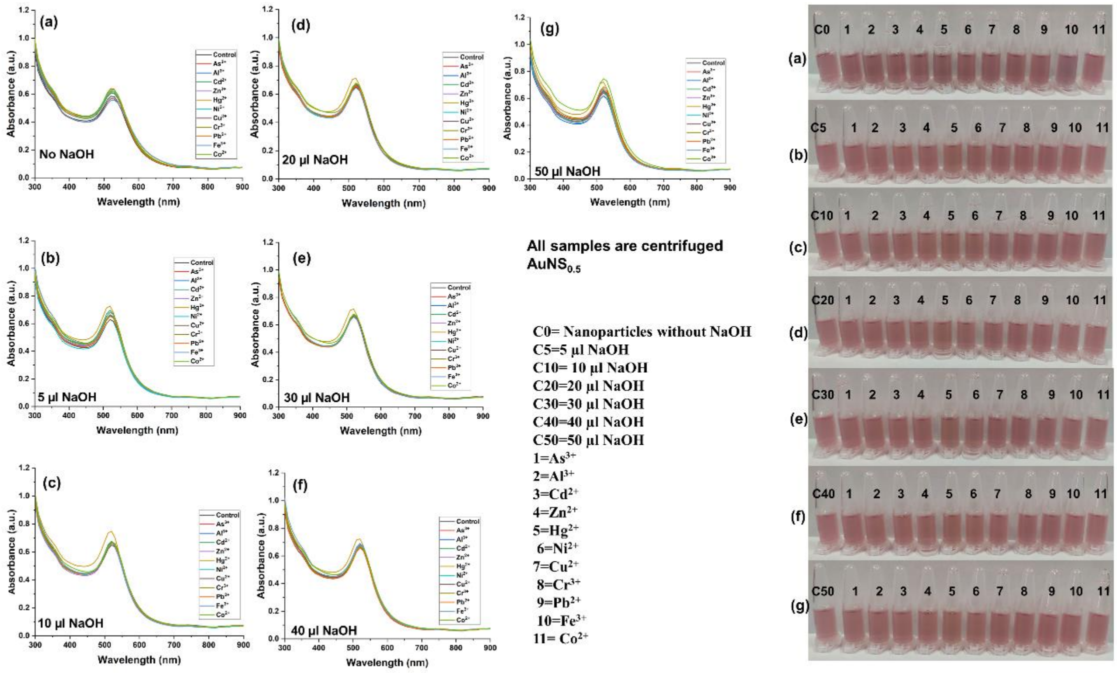

Figure S6: Spectrophotometric and colorimetric detection of heavy metal ions using centrifuged CTAB-AuNS at 0.5 OD in the absence (a) and presence (b-g) of NaOH (5-50 µL).

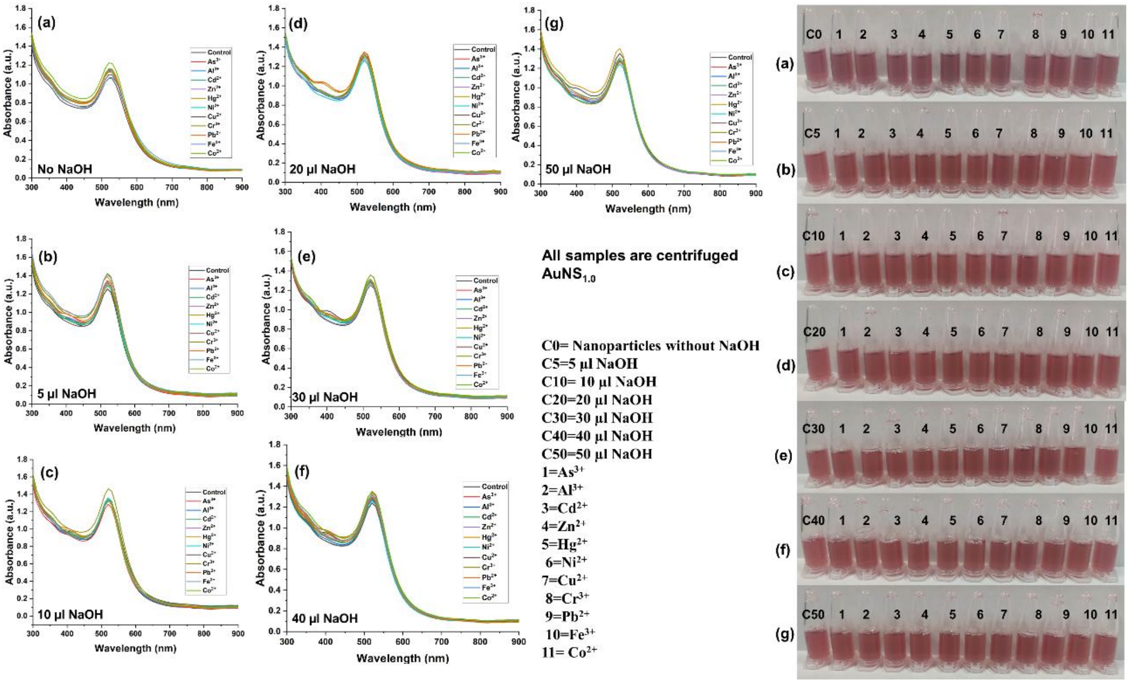

Figure S7: Spectrophotometric and colorimetric detection of heavy metal ions using centrifuged CTAB-AuNS at 1 OD in the absence (a) and presence (b-g) of NaOH (5-50 µL).

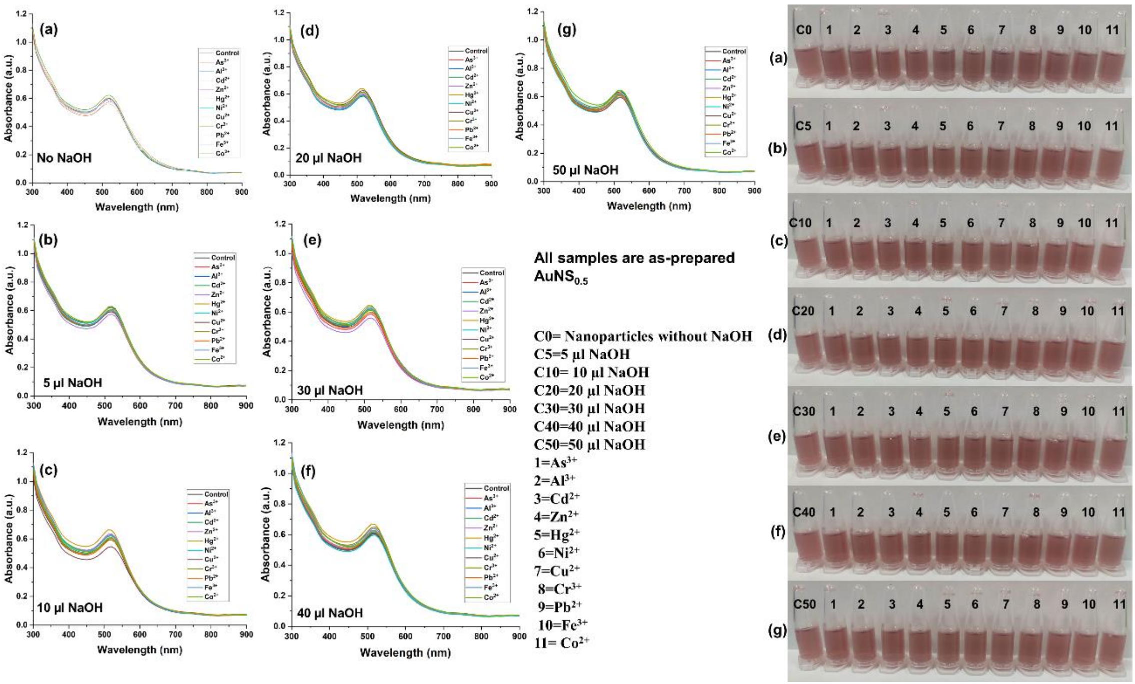

Figure S8: Spectrophotometric and colorimetric detection of heavy metal ions using as-prepared CTAB-AuNS at 0.5 OD in the absence (a) and presence (b-g) of NaOH (5-50 µL).

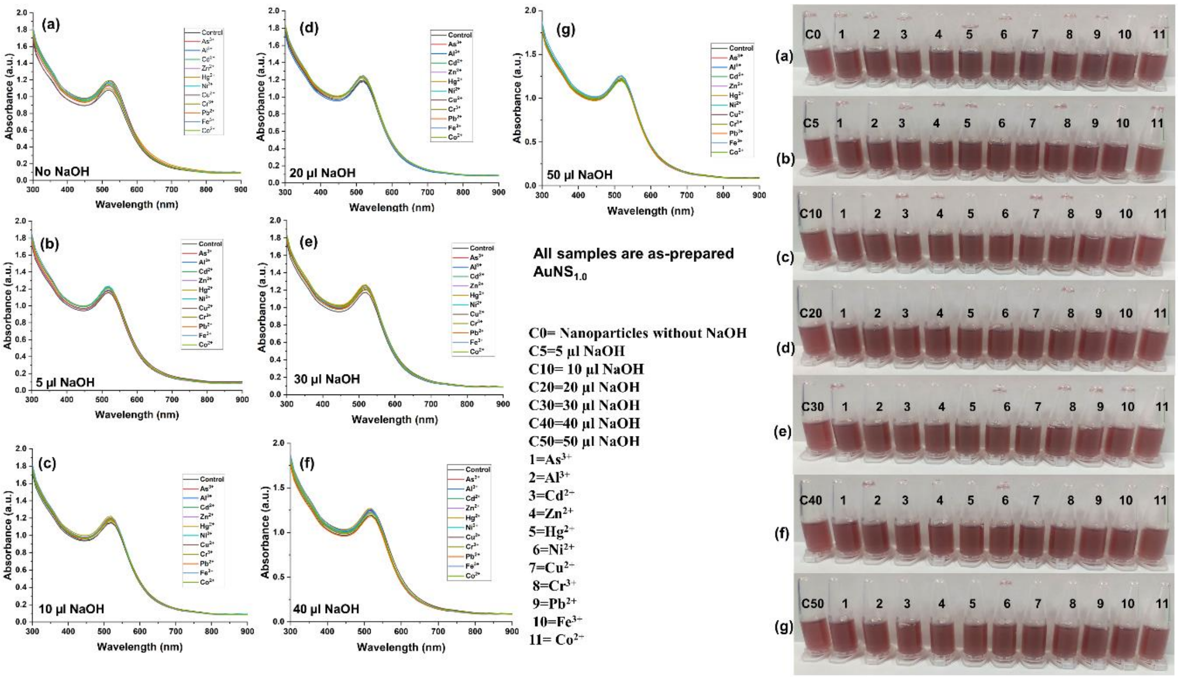

Figure S9: Spectrophotometric and colorimetric detection of heavy metal ions using as-prepared CTAB-AuNS at 1 OD in the absence (a) and presence (b-g) of NaOH (5-50 µL).

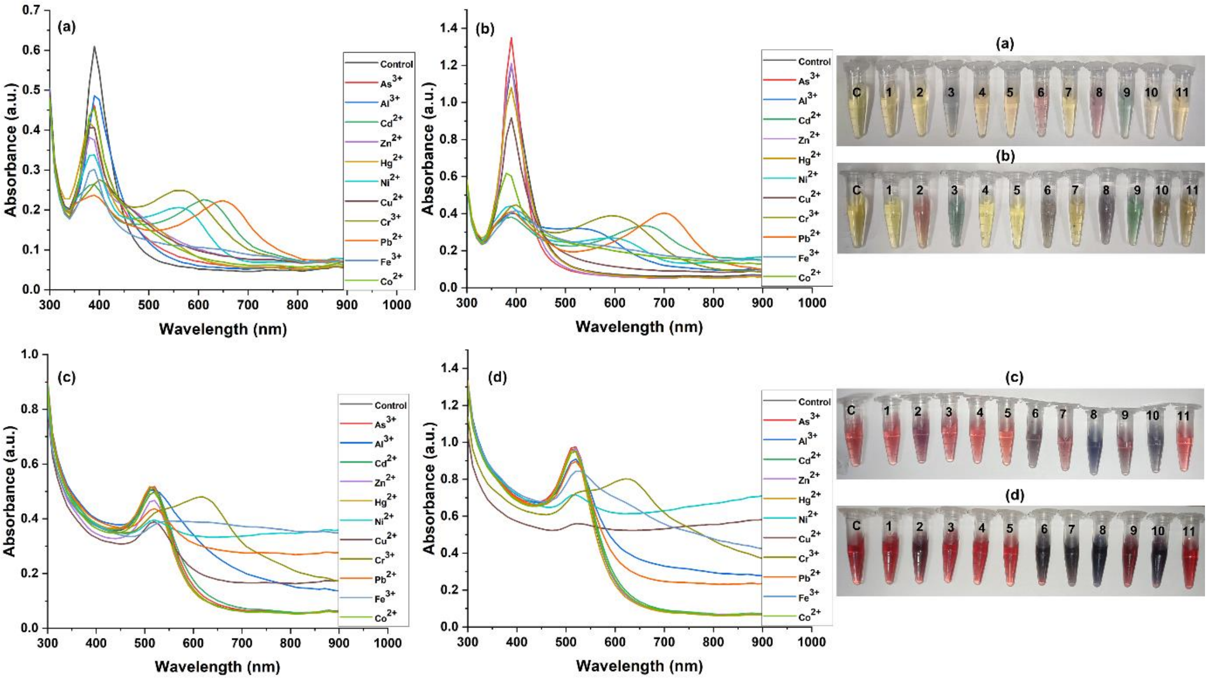

Figure S10: Spectrophotometric and colorimetric detection of heavy metal ions: a) and b) (Bare AgNS at 0.5 and 1 OD), c) and d) (Bare AuNS at 0.5 and 1 OD). The C represents control sample without metal ions in colorimetric images.

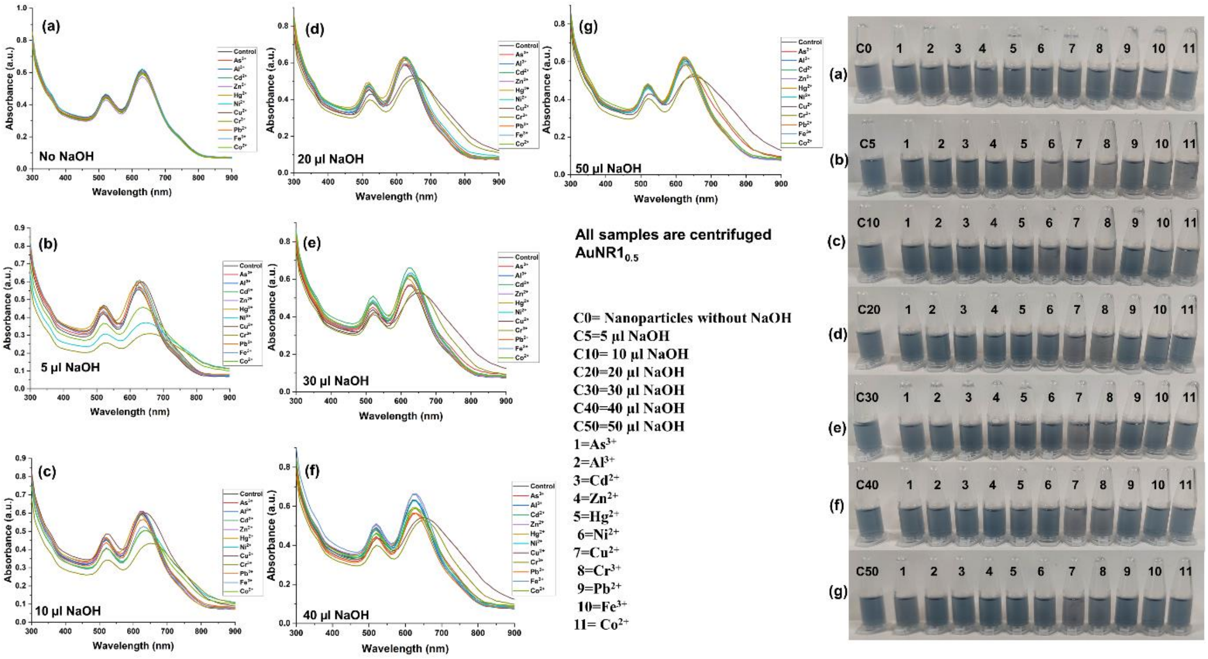

Figure S11: Spectrophotometric and colorimetric detection of heavy metal ions using centrifuged CTAB-AuNR1 at 0.5 OD in the absence (a) and presence (b-g) of NaOH (5-50 µL).

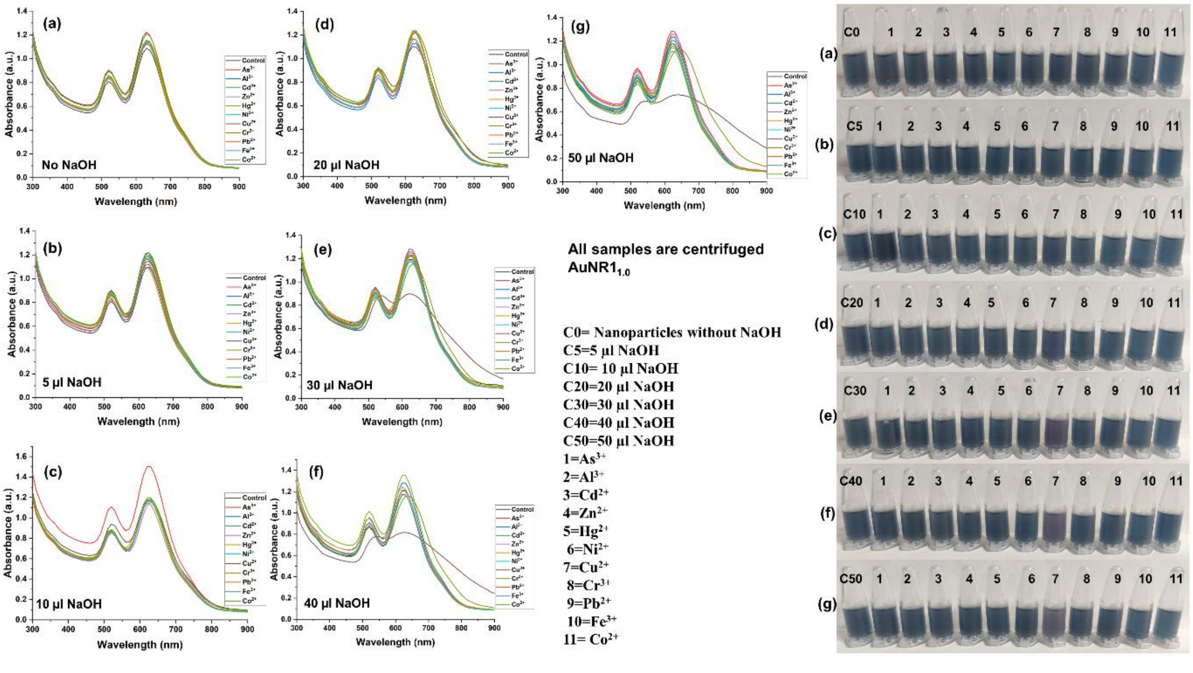

Figure S12: Spectrophotometric and colorimetric detection of heavy metal ions using centrifuged CTAB-AuNR1 at 1 OD in the absence (a) and presence (b-g) of NaOH (5-50 µL).

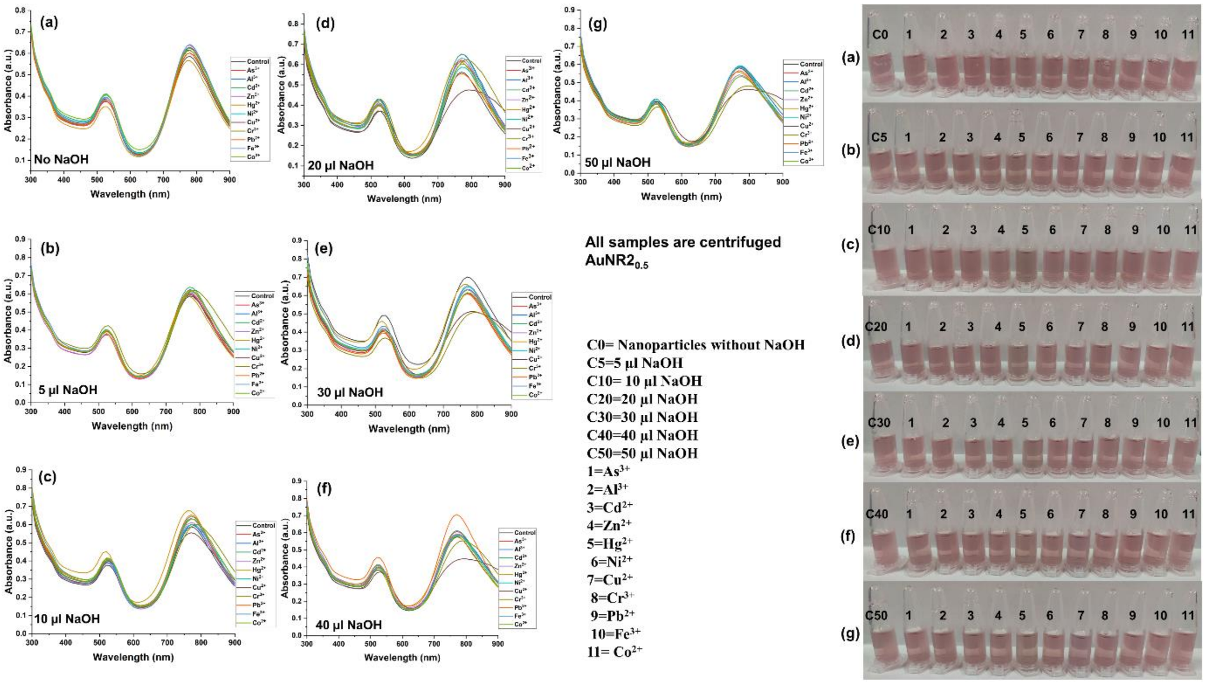

Figure S13: Spectrophotometric and colorimetric detection of heavy metal ions using centrifuged CTAB-AuNR2 at 0.5 OD in the absence (a) and presence (b-g) of NaOH (5-50 µL).

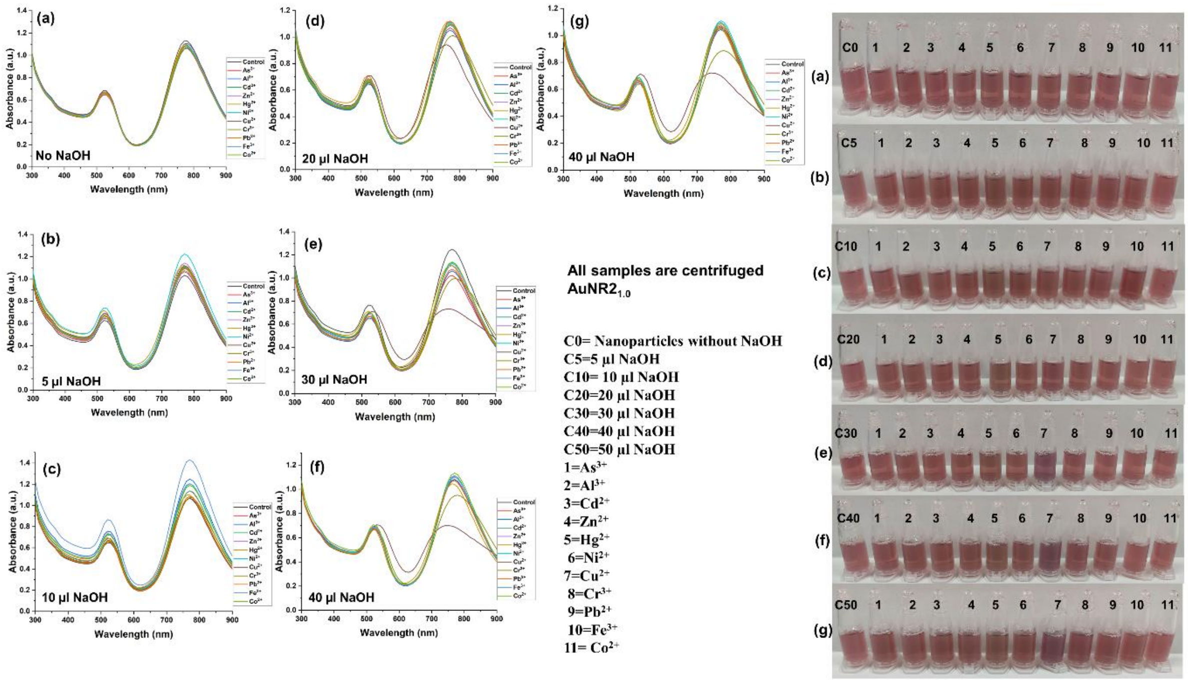

Figure S14: Spectrophotometric and colorimetric detection of heavy metal ions using centrifuged CTAB-AuNR2 at 1 OD in the absence (a) and presence (b-g) of NaOH (5-50 µL).

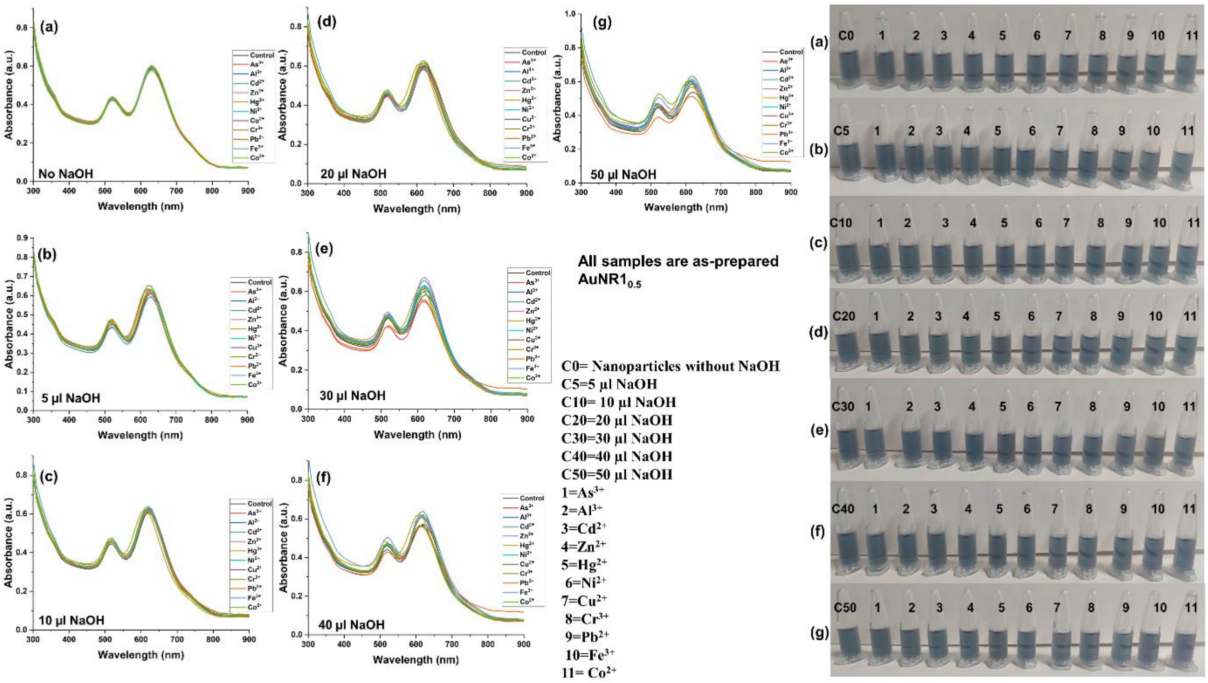

Figure S15: Spectrophotometric and colorimetric detection of heavy metal ions using as-prepared CTAB-AuNR1 at 0.5 OD in the absence (a) and presence (b-g) of NaOH (5-50 µL).

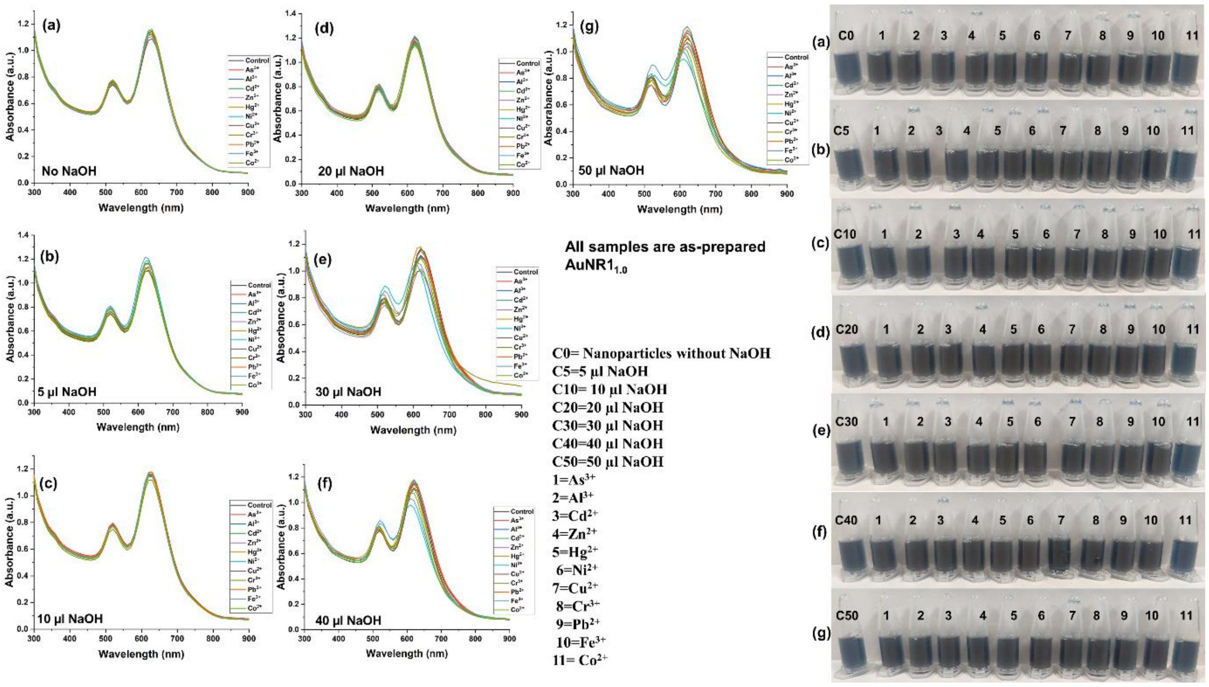

Figure S16: Spectrophotometric and colorimetric detection of heavy metal ions using as-prepared CTAB-AuNR1 at 1 OD in the absence (a) and presence (b-g) of NaOH (5-50 µL).

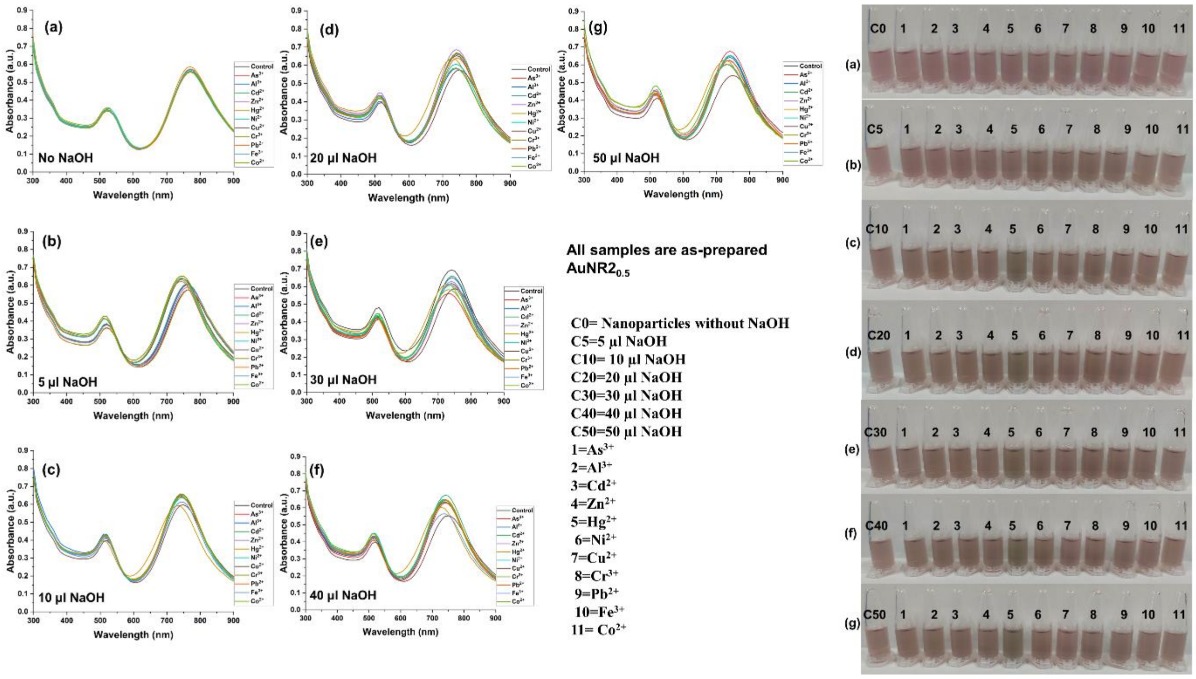

Figure S17: Spectrophotometric and colorimetric detection of heavy metal ions using as-prepared CTAB-AuNR2 at 0.5 OD in the absence (a) and presence (b-g) of NaOH (5-50 µL).

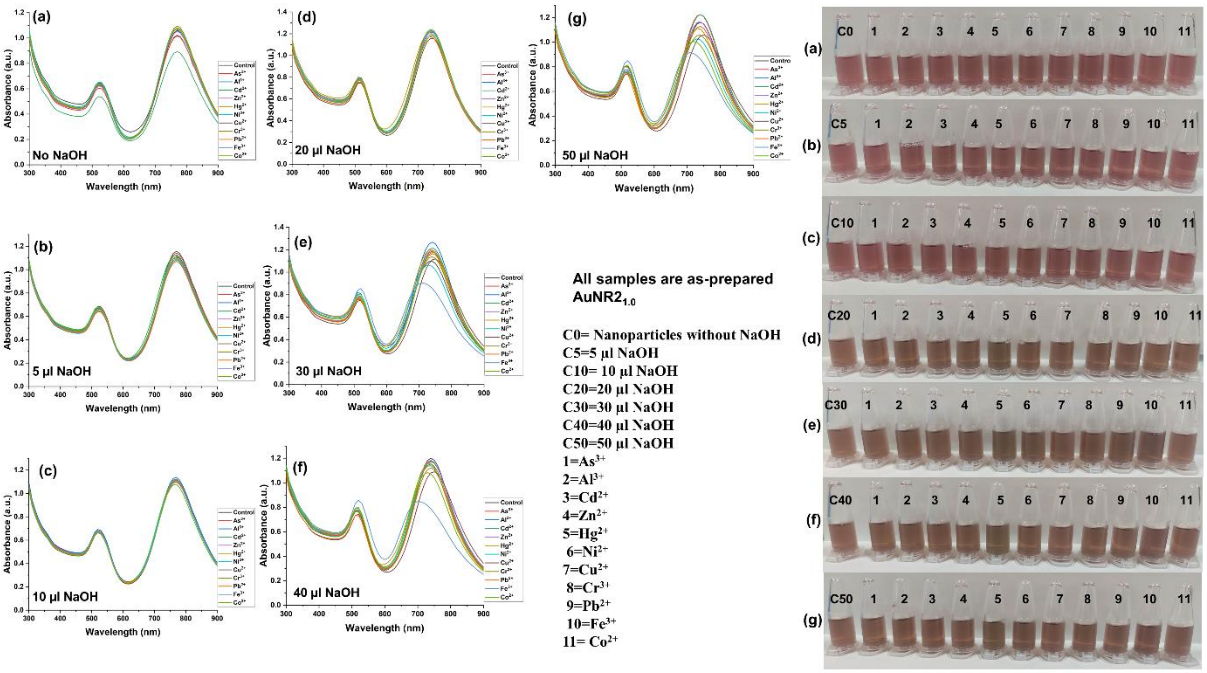

Figure S18: Spectrophotometric and colorimetric detection of heavy metal ions using as-prepared CTAB-AuNR2 at 1 OD in the absence (a) and presence (b-g) of NaOH (5-50 µL).

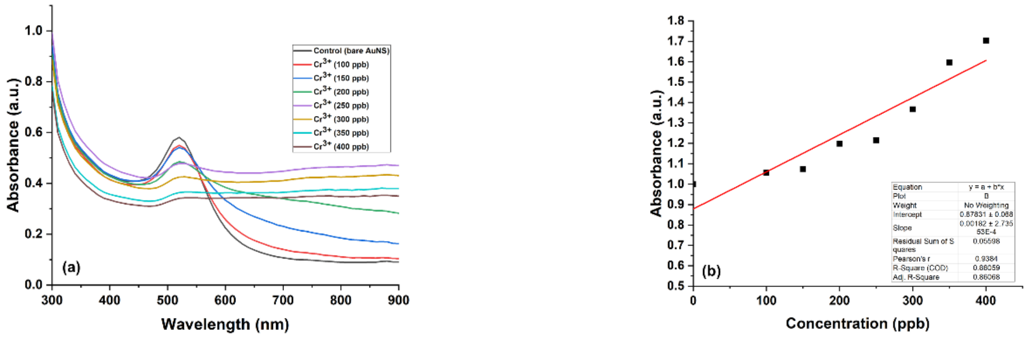

Figure S19: Optical absorbance of (a) bare AuNS with different concentrations of chromium ion and (b) Linear plot for quantification of LOD and LOQ of chromium.

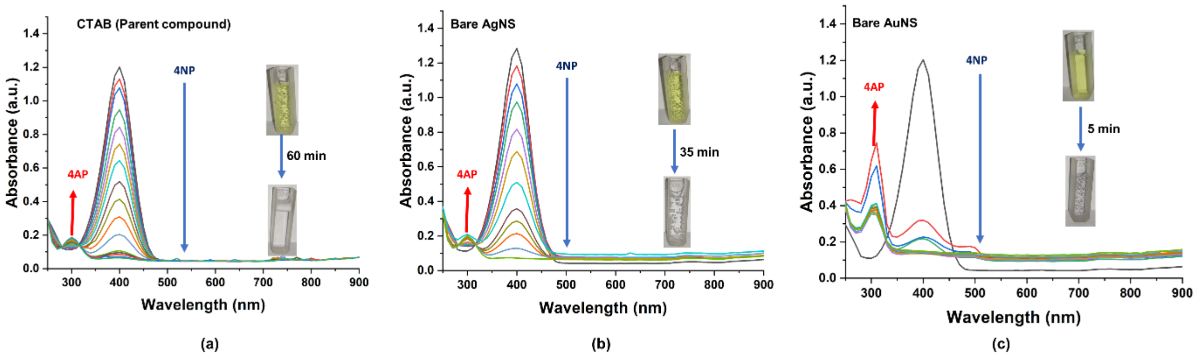

Figure S20: Catalytic degradation of 4-NP using a) CTAB, b) Bare AgNS and c) Bare AuNS.
